# Metabolite, immunocyte phenotype, and lymphoma: a Mendelian randomization study

**DOI:** 10.3389/fimmu.2024.1431261

**Published:** 2024-09-25

**Authors:** Chenyang Fan, Pengying Yuan, Xiangdong Yang, Weifeng Zhang, Xingli Wang, Juan Xie, Jing He, Haijing Chen, Lixiang Yan, Zhexin Shi

**Affiliations:** ^1^ First Teaching Hospital of Tianjin University of Traditional Chinese Medicine, National Clinical Research Center for Chinese Medicine Acupuncture and Moxibustion, Tianjin, China; ^2^ Hospital of University of International Business and Economics, Beijing, China

**Keywords:** immunocyte phenotype, Mendelian randomization, lymphoma, metabolite, intermediation factor

## Abstract

**Background:**

Recent studies have confirmed that metabolites and immunocyte phenotype may be associated with the risk of lymphoma. However, the bidirectional causality between metabolites, immunocyte phenotype, disease risk, and whether immunity is an intermediate mediator between metabolism and lymphoma causality is still unclear.

**Objective:**

To elucidate the causal relationship between metabolites, immune cell phenotypes, and lymphomas, we used two-sample Mendelian randomization (MR) and two-step MR analysis.

**Methods:**

Applying large-scale genome-wide association studies (GWAS) pooled data, we selected 1400 metabolites and 731 immunocyte phenotypes with eight lymphoma subtypes for two-sample bi-directional MR analysis. In addition, we used two-step MR to quantify the proportion of metabolite effects on lymphomas mediated by immunocyte phenotype.

**Results:**

This study yielded a bidirectional causal relationship between 17 metabolites and lymphoma and a bidirectional causal relationship between 12 immunocyte phenotypes and lymphoma. In addition, we found causal associations between metabolites and lymphomas, three groups of which were mediated by immunocyte phenotypes. Among them, CD27 on plasmablast/plasma cell (PB/PC) was a mediator of the positive association of arginine to glutamate ratio with chronic lymphocytic leukemia, with a mediator ratio of 14.60% (95% CI=1.29-28.00%, P=3.17 × 10-2). Natural killer (NK) cells as a percentage of all lymphocytes(NK %lymphocyte) was a mediator of the negative association of X-18922(unknown metabolite) levels with diffuse large B-cell lymphoma, with a mediation proportion of -8.940% (95% CI=-0.063-(-17.800) %, P=4.84 × 10-2). CD25 on IgD- CD24- B cell was the mediator of the positive association between X-24531(unknown metabolite) levels and diffuse large B-cell lymphoma, with a mediation proportion of 13.200% (95% CI=-0.156-26.200%, P=4.73 × 10-2).

**Conclusion:**

In the present study, we identified a causal relationship between metabolites and lymphoma, in which immunocyte phenotypes as mediators are involved in only a minor part. The mediators by which most metabolites affect the risk of lymphoma development remain unclear and require further exploration in the future.

## Introduction

Lymphoma is a group of highly heterogeneous malignant tumors originating in the lymphohematopoietic system (1). It is mainly divided into two categories, Hodgkin’s lymphoma (HL) and non-Hodgkin’s lymphoma (NHL), of which about 85-90% are derived from B cells, while the rest of NHL is derived from T cells or natural killer (NK) cells. B-cell NHL is further classified according to its histology, phenotype, and genetics into diffuse large B-cell lymphoma (DLBCL), follicular lymphoma (FL), mantle cell lymphoma (MCL), marginal zone B-cell lymphoma (MZL), chronic lymphocytic leukemia (CLL), Waldenstrom macroglobulinemia (WM) and others (2). Lymphoma is a malignant tumor characterized by disease at multiple sites throughout the body. Its etiology is complex and difficult to map out. Overall, lymphoma treatment is based on systemic chemotherapy. Despite the proliferation of therapeutic options for lymphoma patients in recent years, the prognosis for lymphoma patients remains worrisome.

Metabolites are intermediate or final products of metabolic reactions, including lipids, amino acids, nucleotides, cofactors, vitamins, carbohydrates, peptides, energy, and several unnamed small molecule metabolites (3). Their levels are influenced by various factors such as genetics, diet, and gut microbes, as well as being associated with disease risk, and are potential therapeutic targets for disease interventions (4). In addition to currently identified blood metabolites, intermetabolite ratios are strongly associated with disease risk (5).

Immunodeficiency has become a recognized risk factor for developing lymphoma (6, 7). However, immunotherapy has demonstrated variable therapeutic efficacy for different types of lymphoma (1). For example, DLBCL responds poorly to immune checkpoint inhibition compared to HL. Therefore, immune targets for various lymphoma types need to be further explored.

Metabolites and immune characteristics appear to be strongly associated with the risk of lymphoma. Recent studies have overturned the previously held belief that “metabolic disorders caused by immune abnormalities lead to cancer” (8–10). “Metabolites influence tumor development by modulating immunity” has become the mainstream view on the relationship between metabolism, immunity, and tumor (11, 12). This conclusion is not only applicable to solid tumors but also to hematological tumors (13).

We can verify the causal relationship between metabolites, immune characteristics, and the risk of developing lymphoma through randomized controlled trials (RCT). However, because metabolite and immunocyte phenotypes are affected by real-world confounding factors, it is difficult to distinguish the sequential causality of the two in the human body. At this point, we can conduct the study through Mendelian randomization (MR). MR refers to Mendel’s second law, which states that alleles segregate independently during reproduction, combine randomly during hybridization, and are passed on to offspring with equal probability (14). Therefore, it is generally assumed that this genetic variation is randomly distributed in the population and is independent of environmental or lifestyle factors (15). Genetic variation can thus be used as an instrumental variable (IV) to mimic the process of randomly assigning treatment factors to experimental and control groups in RCT studies, thereby reducing the influence of confounding factors in the results of these studies and avoiding the difficulty of determining the temporal order of antecedents and consequences in traditional observational studies (16).

In the present study, we verified the association between metabolites, immunocyte phenotypes, and lymphomas (including HL, DLBCL, FL, MCL, MZL, WM, CLL, and Mature T/NK-cell lymphomas) using MR. We also verified whether immunity mediates metabolic abnormalities leading to lymphomas. Finally, we also reverse-validated the trends of metabolites and immunocyte phenotypes that accompanied the development of lymphomas.

## Methods

### Study design

**Figure 1:**
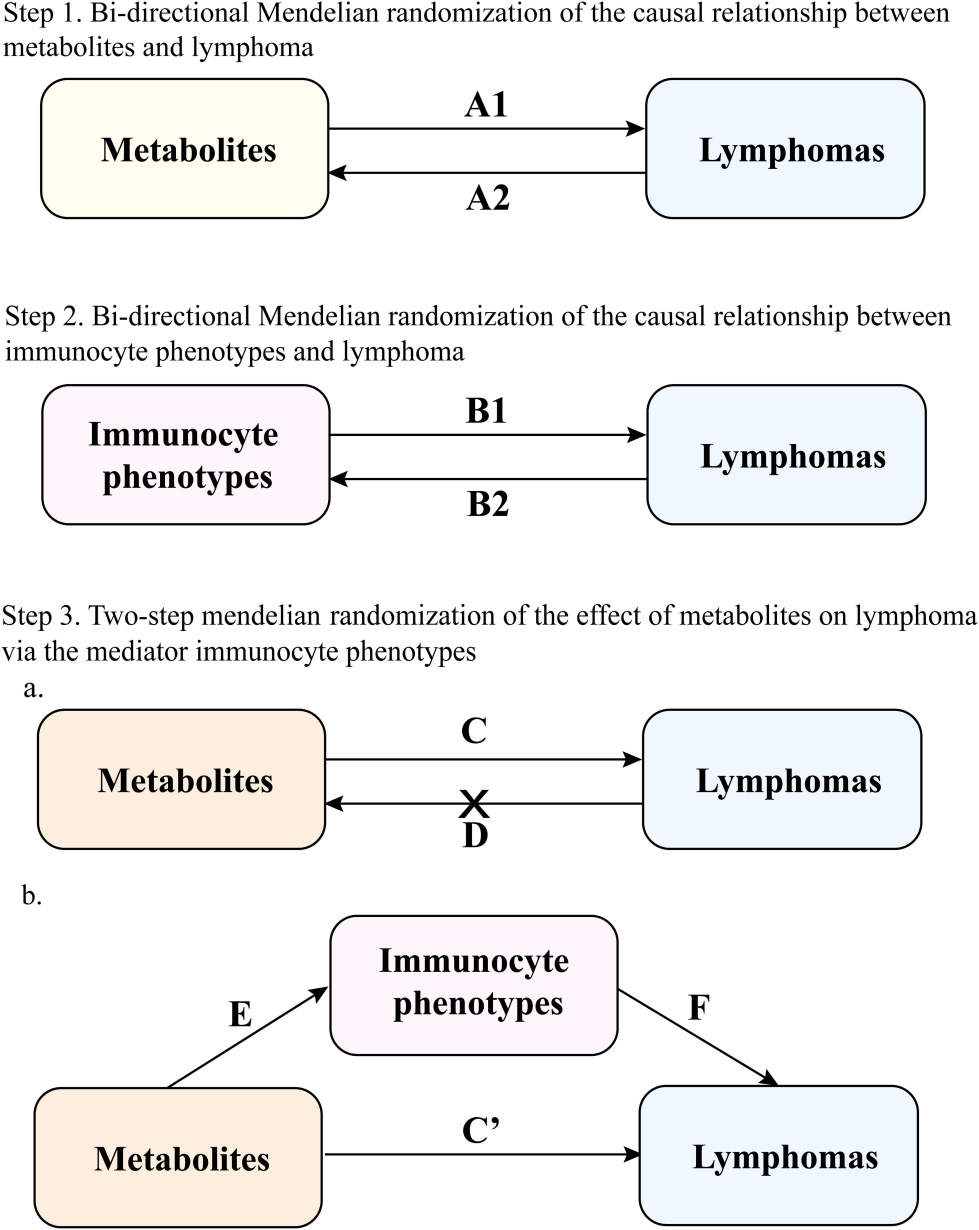
Study overview. A1 represents the positive effect of metabolites on lymphoma; A2 represents the reverse effect of lymphoma on metabolites; B1 represents the positive effect of immunocyte phenotypes on lymphoma; B2 represents the reverse effect of lymphoma on immunocyte phenotypes; C represents the total effect of metabolites on lymphoma; D represents the reverse total effect of lymphoma on metabolites, and screened for metabolites with negative reverse MR; in Step 3 the mediating effect (E represents the effect of the metabolite on the immunocyte phenotypes and F represents the effect of the immunocyte phenotypes on the lymphoma) was calculated as (E × F). The direct effect C’ was calculated as (total effect C - mediating effect E × F).

As shown in **Figure 1**, this study consists of three parts. First, the first two parts verified the bidirectional causal relationship between metabolites, immunocyte phenotypes, and lymphomas using bi-directional MR, respectively. The third part verified the mediating effect of immunocyte phenotypes between metabolites and lymphoma using two-step MR.

### Data source

The GWAS data for metabolites are derived from the most recent summary of genetic data for the European population. The GWAS data include 1091 plasma metabolites and 309 metabolite ratios. The 1091 plasma metabolites include 850 metabolic constituents already confirmed in the eight super pathways (i.e., lipids, amino acids, probiotics, nucleotides, cofactors and vitamins, carbohydrates, peptides, and energy), with the remaining 241 classified as unknown molecules (3).

We downloaded all single nucleotide polymorphisms (SNPs) associated with human immunocyte phenotypes as IVs in the IEU Open GWAS project (https://gwas.mrcieu.ac.uk/). It is a 22 million variant pair of RNA gene sequencing and genotyping data on immunocyte phenotypes from 3757 European participants. to explore the complex genetic regulation of immune cells in autoimmune diseases. The total of 731 immunocyte phenotypes included absolute cell counts (n=118), relative counts (n=192), median fluorescence intensities (MFIs) of surface antigens (n=389), and morphological parameters (n=32) (17).

We obtained all GWAS pooled data (controls excluding all cancers) with the eight lymphoma subtypes from the FinnGen Consortium database (version R10). All data are of European origin. Detailed information on participants, genotype platforms, and statistical analysis protocols is available on the FinnGen website (https://www.FinnGen.fi/en/). Detailed information is available in an **Additional File** (**Supplementary Table S1**).

### Selecting genetic instruments

The genetic instruments employed had to fulfill three assumptions (15): 1) the genetic variants should be strongly associated with the exposure, 2) the genetic variants should not be associated with any potential confounding factors, and 3) the genetic variants should not affect the outcome independently of exposure. We excluded variants with minor allele frequencies <0.01 in the GWAS dataset. We used a strict r^2^ <0.001 threshold, a 10,000 kb window, and a clustering method with P <1 × 10^-5^. Notably, because of the limited number of SNPs in WM, P was chosen as a threshold of 5 × 10^-5^ when WM was used as an exposure factor. To ensure consistency, we harmonized the effects of SNPs on both exposure and outcome by aligning the beta values to the identical alleles. F statistics were calculated to evaluate the strength of instrumental variables, with F > 10 indicating no weak instrumental variable bias, and instrumental variables with F < 10 were excluded.

### MR analysis

#### Primary analysis

First, we used two-sample bidirectional MR (corresponding to A1 and B1 in **Figure 1**) to demonstrate the causal associations between metabolites, immunocyte phenotypes, and eight lymphoma subtypes. The inverse variance weighted (IVW) was used as the primary method to analyze the causal association between exposure factors and outcome variables (16). We validate the results using MR-Egger and Weighted median. In contrast to IVW, the MR-Egger method accounts for the presence of an intercept term (18). The weighted median provides consistent estimates if at least 50% of the valid instrumental variables are present in the analysis (19). According to the third hypothesis of MR analysis, instrumental variables must be associated with outcomes only through exposure. Therefore, this study used the MR-Egger regression method to assess potential horizontal pleiotropy through intercept term. Additionally, this study utilized Simple mode versus Weighted mode analysis.

#### Bi−directional causality analysis

We performed to verify the reverse causality of lymphoma with metabolites and immunocyte phenotypes. We chose lymphoma as an exposure factor and metabolites and immunocyte phenotypes as outcome factors (corresponding to A2 and B2 in **Figure 1**).

#### Mediation analysis

**Figure 2:**
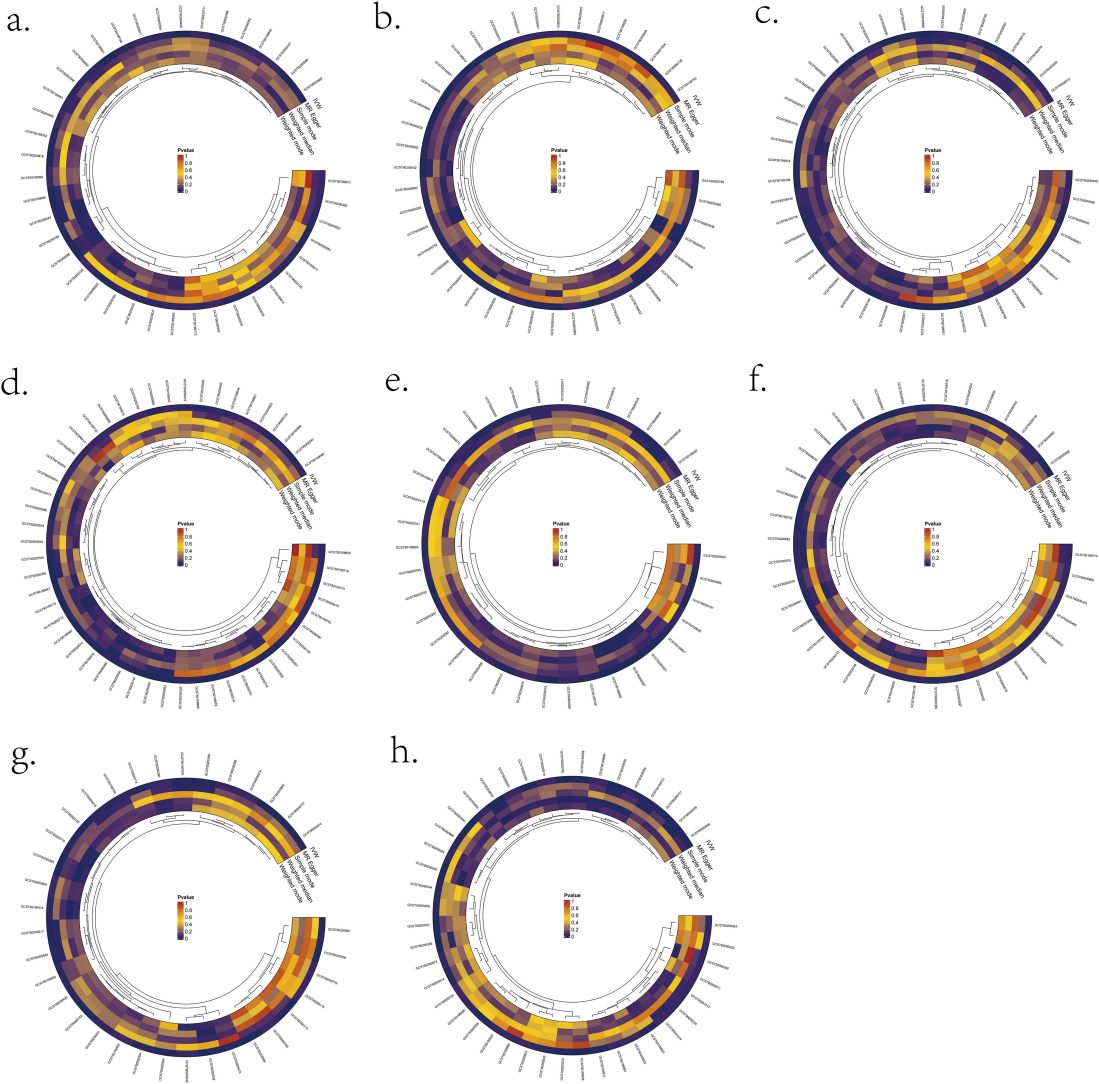
Significant MR results for the relationship between 1400 metabolites and eight types of Lymphomas. **(A)** MR analysis results between metabolites and DLBCL. **(B)** MR analysis results between metabolites and FL. **(C)** MR analysis results between metabolites and MCL. **(D)** MR analysis results between metabolites and CLL. **(E)** MR analysis results between metabolites and MTCL. **(F)** MR analysis results between metabolites and HL. **(G)** MR analysis results between metabolites and WM. **(H)** MR analysis results between metabolites and MZL.

We designed a two-step MR for mediation analysis to verify whether immunocyte phenotypes are intermediate mediators of the causal relationship between metabolites and lymphoma. This study yielded a positive MR total effect for metabolites and lymphoma (corresponding to C in **Figure 1**). Reverse MR analysis was then performed to derive metabolites with negative MR correlations as exposure data in the following analyses (corresponding to D in **Figure 1**). Next, this study yielded two overall effects, an indirect effect through mediators and a direct effect without mediators (20). The total effect of metabolites on lymphomas was divided into (1) the direct effect of metabolites on lymphomas (corresponding to C’ in **Figure 1**) and (2) the mediated effect of metabolites mediated through a mediator (corresponding to E×F in **Figure 1**). The mediated effect divided by the total effect is the mediated proportion (corresponding to (E × F)/C in **Figure 1**). We applied the delta method to calculate 95% confidence intervals (18).

#### Sensitivity analysis

The direction of MR was verified using MR Steiger to exclude the effect of reverse causality. The results were tested for heterogeneity using Cochran’s Q test, with P<0.05 indicating the presence of heterogeneity and P>0.05 indicating the absence of heterogeneity (20). Horizontal pleiotropy was analyzed using the MR-Egger intercept method. The leave-one-out analysis was used to analyze whether a single SNP affected the MR results. Possible horizontal pleiotropy was examined by looking at the symmetry of the funnel plot to gauge the reliability of the current MR analysis. Outliers were analyzed with the Radial package, and the MR-PRESSO method was used to test the effect of outliers on the results (21).

## Results

### Bi-directional association of metabolites with lymphoma

We validated bi-directional MR between 1400 metabolite metrics and eight lymphomas (**Figure 2**). IVW results showed that two metabolites were bi-directionally associated with DLBCL: X-11632(unknown metabolite) level and Phosphate to the 2’-deoxyuridine ratio (**Supplementary Table S1**). Four metabolites were bi-directionally associated with FL in this study: kynurenine levels, 1-methylxanthine levels, Dihydroferulate levels, and 2’-o-methylcytidine levels (**Supplementary Table S2**). Four metabolites were bi-directionally associated with MCL in this study: octanoylcarnitine (c8) levels, linoleate (18:2n6) levels, X-15728(unknown metabolite) levels, and Arachidonate (20:4n6) to linoleate (18:2n6) ratio (**Supplementary Table S3**). In this study, three metabolites were bi-directionally associated with CLL: Palmitate (16:0) to myristate (14:0) ratio, Glucose to maltose ratio, and Adenosine 5’-diphosphate (ADP) to glycerol 3- phosphate ratio (**Supplementary Table S5**). There was one metabolite that was bi-directionally associated with Mature T/NK-cell lymphomas (MTCL) in this study: the mannose to N-acetylglucosamine to N-acetylgalactosamine ratio (**Supplementary Table S6**). Three metabolites were bi-directionally associated with HL in this study: propionylglycine levels, X-21258(unknown metabolite) levels, and Adenosine 5’-monophosphate (AMP) to inosine 5’-monophosphate (IMP) ratio (**Supplementary Table S7**). There was no bi-directional correlation between WM, MZL, and metabolites (**Supplementary Tables S4**/**S8**).

### Bi-directional association of immunocyte phenotypes with lymphoma

**Figure 3:**
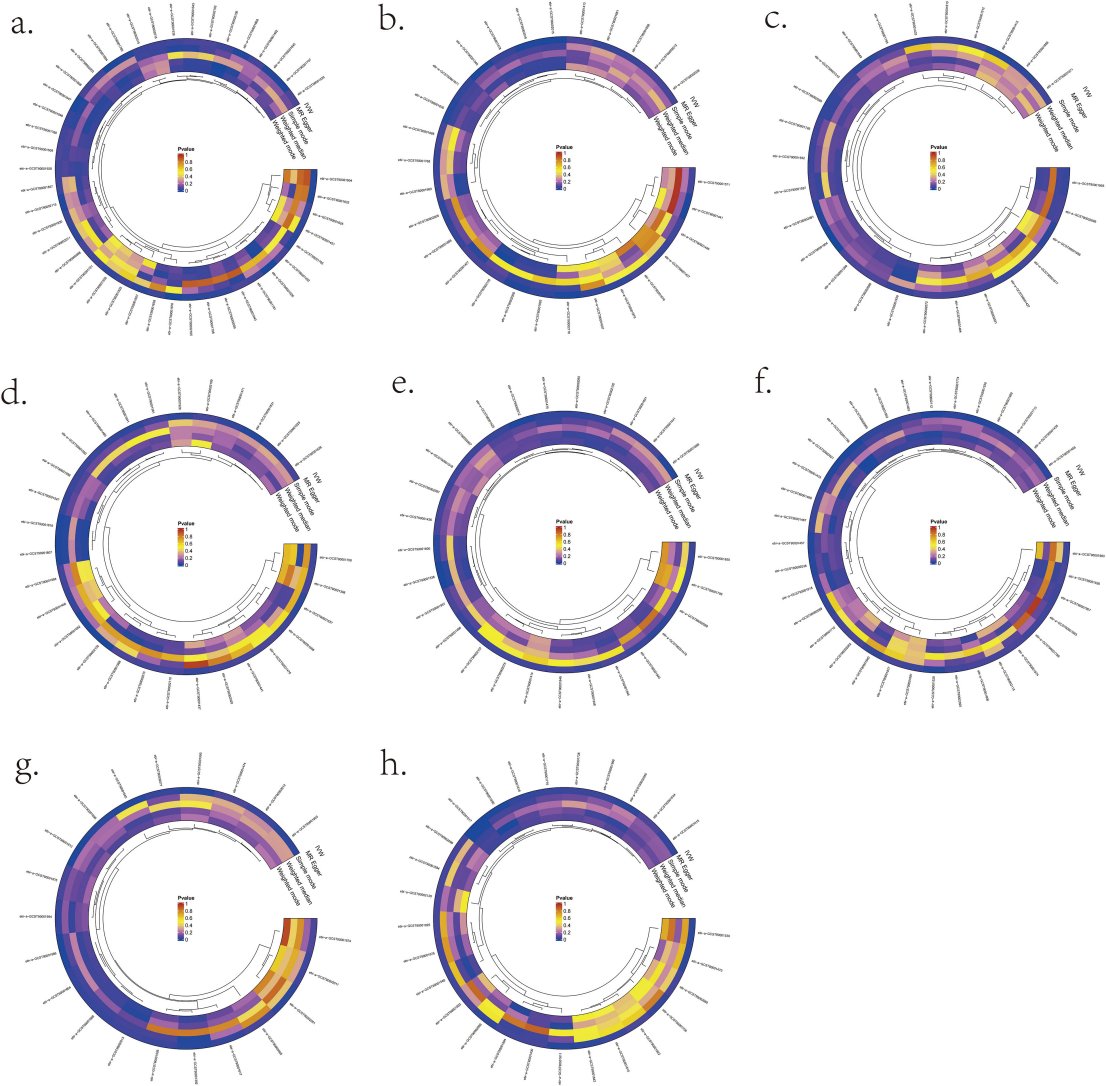
Significant MR results for the relationship between 731 immunocyte phenotypes and eight types of Lymphomas. **(A)** MR analysis results between immunocyte phenotypes and DLBCL. **(B)** MR analysis results between immunocyte phenotypes and FL. **(C)** MR analysis results between immunocyte phenotypes and MCL. **(D)** MR analysis results between immunocyte phenotypes and CLL. **(E)** MR analysis results between immunocyte phenotypes and MTCL. **(F)** MR analysis results between immunocyte phenotypes and HL. **(G)** MR analysis results between immunocyte phenotypes and WM. **(H)** MR analysis results between immunocyte phenotypes and MZL.

We validated bidirectional MR between 731 immunocyte phenotypes and eight lymphomas (**Figure 3**). The most prominent IVW results showed that six immunocyte phenotypes were bi-directionally associated with DLBCL: T cell as a percentage of all lymphocytes (T cell %lymphocyte), CD8br as a percentage of all leukocytes (CD8br %leukocyte), B cell as a percentage of CD3- lymphocytes (B cell %CD3- lymphocyte), NK cells as a percentage of CD3- lymphocytes (NK %CD3- lymphocyte), NK cells as a percentage of all lymphocytes (NK %lymphocyte), and CD19 on CD20- (**Supplementary Table S1**). Two immunocyte phenotypes were bi-directionally associated with FL in the present study: the CD28 on CD39+ secreting Treg and the CC chemokine receptor 2 (CCR2) on CD14+ CD16+ monocyte (**Supplementary Table S2**). In the present study, one immunocyte phenotype was bi-directionally associated with MCL: CD25 on transitional (**Supplementary Table S3**). Two immunocyte phenotypes were bi-directionally associated with WM from this study: the CX3CR1 on CD14- CD16- and CD45 on basophil (**Supplementary Table S4**). In the present study, three immunocyte phenotypes were bi-directionally associated with CLL: Unswitched memory B cell Absolute Count (Unsw mem AC), CD86+ plasmacytoid Dendritic Cell Absolute Count (DC AC) and CD127 on CD28+ CD45RA- CD8br (**Supplementary Table S5**). In this study, one immunocyte phenotype was bi-directionally correlated with MTCL: CD4 Treg %CD4 (**Supplementary Table S6**). There was no bi-directional correlation between HL, MZL, and immune cell phenotypes (**Supplementary Table S7**/**S8**).

### Role of immunocyte phenotypes in mediating the effect of metabolites on lymphomas

**Figure 4:**
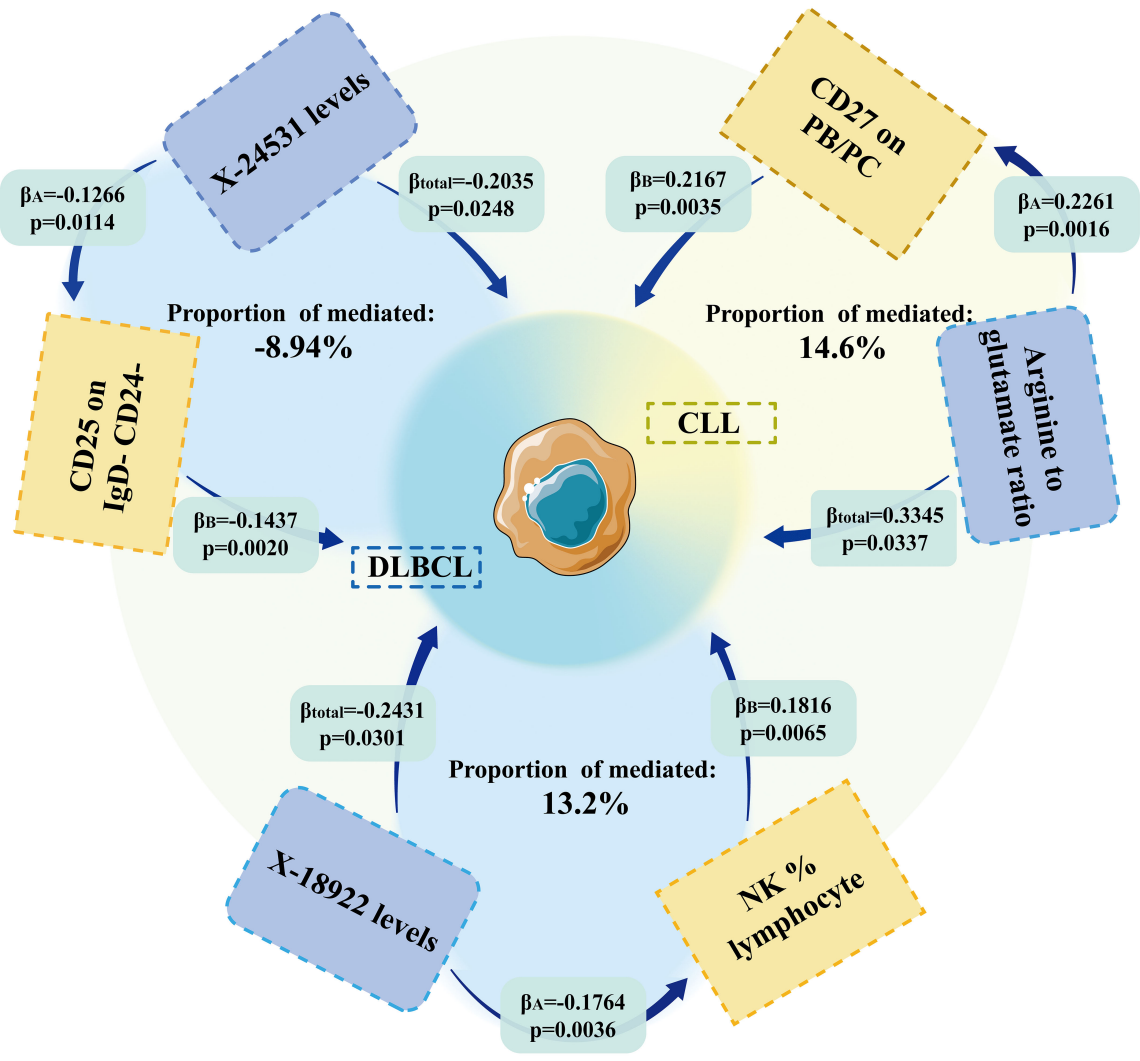
MR results showed that immune cell phenotype was an essential mediator of the causal relationship between metabolites and lymphoma.

**Figure 5:**
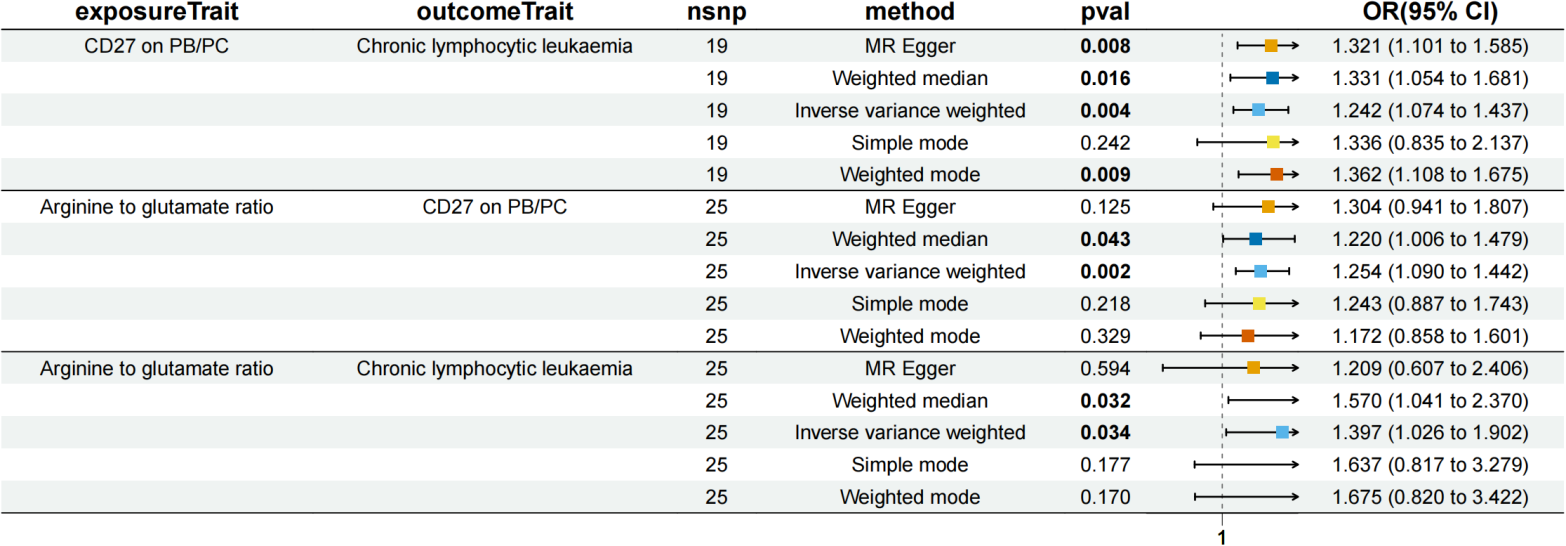
Forest plot to visualize the causal effects of CD27 on PB/PC with arginine to glutamate ratio and chronic lymphocytic leukaemia.

We analyzed whether 731 immunocyte phenotypes were causal mediators between 1400 metabolites and eight phenotypic lymphomas. The results revealed three significant groups of mediating correlations between metabolites, immunocyte phenotypes, and lymphomas (**Table 1**) (**Figure 4**). Among them, CD27 on Plasma Blast-Plasma Cells (CD27 on PB/PC) can mediate the causal relationship between arginine to glutamate ratio and CLL (**Figure 5**). Arginine to glutamate ratio was negatively associated with CD27 on PB/PC (β=0.226,95% CI=0.086-0.336, P=1.56 × 10^-3^), which subsequently led to an increased risk of CLL with a mediation ratio of 14.60% (95% CI=1.29- 28.00%, P=3.17 × 10^-2^) (**Supplementary Table S9**).

**Table 1:** Mendelian randomization probes the mediating role of immunocyte phenotype in the association between metabolites and lymphoma.

Metabolite	Immunity trait	Outcome	Total Effect	Direct Effect A	Direct Effect B	Mediated effect		Mediated proportion
			β (95% Cl)	β (95% Cl)	β (95% Cl)	β (95% Cl)	*P*-value	
Arginine to glutamate ratio	CD27 on PB/PC	Chronic lymphocytic leukaemia	0.3345(0.0258, 0.6432)	0.2261(0.0860, 0.3662)	0.2167(0.0712, 0.3622)	0.0490(0.0043, 0.0937)	0.0317	14.600% (1.290%, 28.000%)
X-18922 levels	NK %lymphocyte	Diffuse large B-cell lymphoma	-0.2035(-0.3811, -0.0258)	-0.1266(-0.2247, -0.0285)	-0.1437(-0.2351, -0.0524)	0.0182(0.0001, 0.0363)	0.0484	-8.940% (-0.063%, -17.800%)
X-24531 levels	CD25 on IgD- CD24-		-0.2431(-0.4630, -0.0234)	-0.1764(-0.2949, -0.0578)	0.1816(0.0508, 0.3125)	-0.0320(-0.0637, -0.0004)	0.0473	13.200% (0.156%, 26.200%)

Total effect: indicates the effect of metabolite on lymphoma; direct effect A: the effect of metabolite on immunocyte phenotype; direct effect B: the effect of immunocyte phenotype on lymphoma; mediation effect: the effect of metabolite on lymphoma through affecting immunocyte phenotype. The total effect, direct effect A, and direct effect B were derived using the inverse-variance weighted method; the mediation effect was derived using the delta method. All statistical tests were 2-sided. P<0.05 was considered significant.

**Figure 6:**
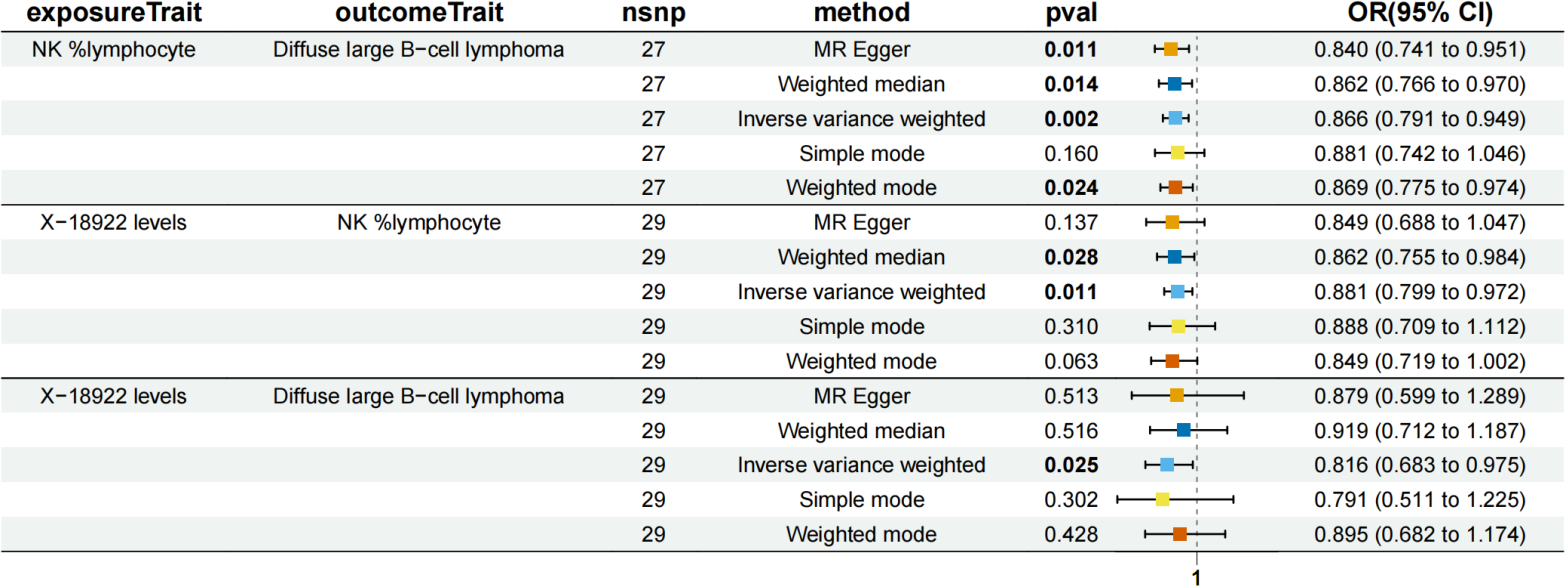
Forest plot to visualize the causal effects of NK %lymphocyte with X-18922 levels and diffuse large B-cell lymphoma.

NK %lymphocyte mediated the causal relationship between X-18922 (unknown metabolite) levels and DLBCL (**Figure 6**). X-18922 levels were negatively correlated with NK %lymphocyte (β=-0.126, 95%CI=-0.225-(-0.029) %, P=1.14 ×10^-2^), which subsequently led to an increased risk of DLBCL with a mediation ratio of -8.940% (95% CI=-0.063-(-17.800) %, P=4.84 × 10^-2^) (**Supplementary Table S9**).

**Figure 7:**
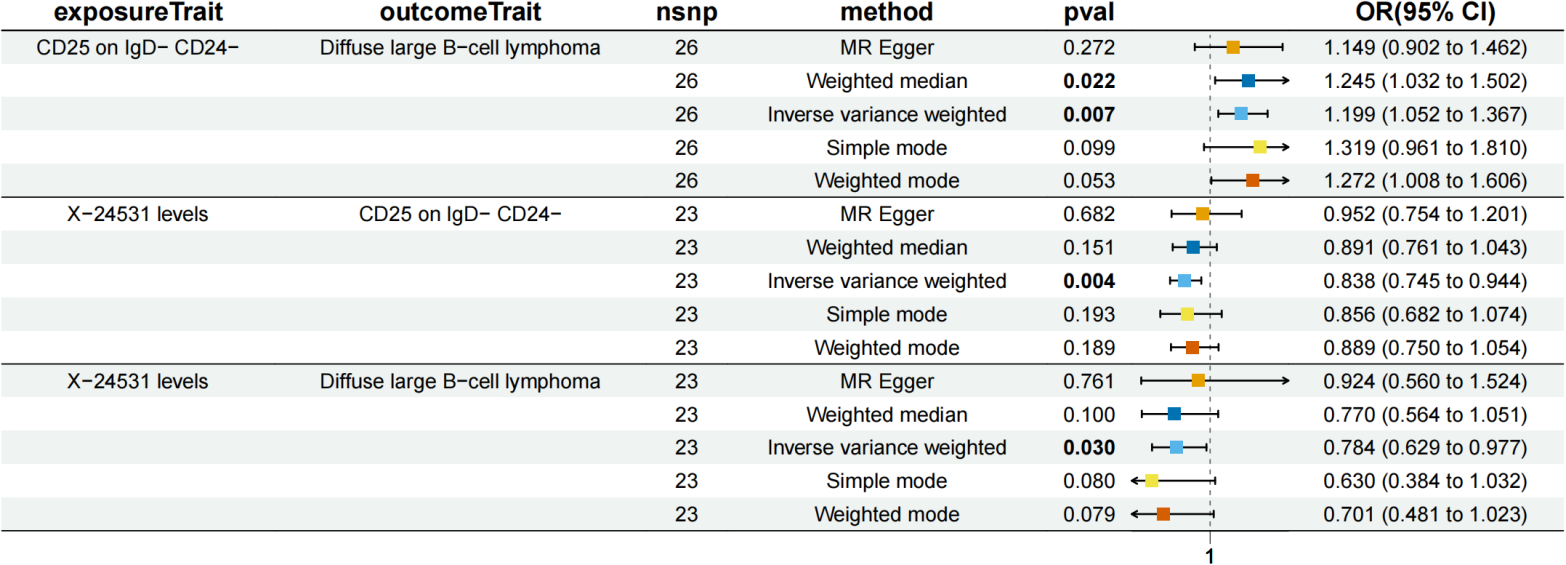
Forest plot to visualize the causal effects of CD25 on IgD- CD24- B cell with X-24531 levels and diffuse large B-cell lymphoma.

CD25 on IgD- CD24- B mediated the causal relationship between X-24531 (unknown metabolite) levels and DLBCL (**Figure 7**). X-24531 levels were negatively correlated with CD25 on IgD- CD24- B cell (β=-0.176,95%CI=-0.295-(- 0.058), P=3.55 × 10^-3^), which subsequently led to an increased risk of DLBCL with a mediation ratio of 13.200% (95% CI=-0.156-26.200%, P=4.73 × 10^-2^) (**Supplementary Table S9**).

### Sensitivity analyses

According to the MR-Egger regression intercept approach, genetic pleiotropy did not bias the results.MR-PRESSO analysis revealed that two immunocyte phenotypes (T cell %lymphocyte and CD8br %leukocyte) were horizontally pleiotropic for DLBCL (P < 0.05), and one immunocyte phenotype (CCR2 on CD14+ CD16+ monocyte) was horizontally pleiotropic for FL (P < 0.05, (**Supplementary Table S10**), and therefore the above results were excluded. MR-PRESSO analysis demonstrated no horizontal pleiotropy in the other parts of the MR study (P > 0.05, (**Supplementary Table S10**). Cochran’s Q test showed significant heterogeneity of four immunocyte phenotypes (T cell %lymphocyte, CD8br %leukocyte, B cell % CD3- lymphocyte, and NK %CD3- lymphocyte) for DLBCL (P < 0.05) and one immunocyte phenotype (CCR2 on CD14+ CD16+ monocyte) for FL (P > 0.05, (**Supplementary Table S10**). The other sections did not show significant heterogeneity in Cochran’s Q test (P > 0.05, (**Supplementary Table S10**).

The “leave-one-out” analysis proved that the MR results were reliable (**Supplementary Figures S11**, **S1-6**). The scatter plot shows the overall effect of metabolites on lymphoma (**Supplementary Figures S11**, **S7-12**). Finally, the forest plot shows the causal relationship between metabolites and lymphoma (**Supplementary Figures S11**, **S13-18**).

## Discussion

Metabolites are small molecular compounds into which metabolism converts nutrients. It is also essential for all types of cells in the body to maintain their functions (22). It has been known that the metabolic pathways of tumor cells are different from those of other cells. According to the “Warburg effect,” glycolysis is preferred over oxidative phosphorylation in tumor cells (23). Glycolysis will supply adenosine triphosphate to tumor cells more rapidly, providing a faster energy supply for unlimited proliferation of tumor cells (24). Of course, the metabolism of lymphoma cells also follows the “Warburg effect,” and this unique metabolism inevitably makes metabolites a risk factor for lymphoma progression.

Immunity has always been a critical challenge in cancer risk research (25). Several immunologic drugs have been approved for clinical use, including various immunosuppressants and chimeric antigen receptor-T cell therapies (6). Among them, lymphomas are malignant tumors originating from the cells of the immune system (26). It is even more inextricably linked to immunization. However, we still do not know which immune phenotype is involved in the disease process of lymphoma.

Notably, according to recent studies, tumor cells compete for metabolic energy through a unique metabolic approach, which affects immune cell metabolism and inhibits the anti-tumor activity of immune cells (11). Lymphoma is closely related to both. Exploring the cascade effect between metabolites, immunophenotypes, and lymphomas would provide a new basis for new drug development and clinical therapeutic direction. The treatment effect will be twice as favorable with half the effort. Therefore, in this study, we applied MR analysis to verify the causal relationship between 1400 metabolites, 731 immunocyte phenotypes, and eight types of lymphomas (DLBCL, FL, MCL, MZL, CLL, WM, MTCL, and HL). And whether immunocyte phenotypes mediate the causal relationship between metabolites and the risk of lymphoma development.

Unknown metabolite X-11632 levels and a high phosphate to 2’-deoxyuridine ratio decreased the risk of DLBCL. High values of immunocyte phenotype B cell % CD3- lymphocyte increased the risk of DLBCL, and high values of NK %CD3- lymphocyte, NK %lymphocyte, and CD19 on CD20- decreased the risk of DLBCL. NK cells are derived from bone marrow hematopoietic stem cells and are essential components of the intrinsic immune system (27). NK cells not only act as killer cells to inhibit tumors, but NK cells can also use interferon gamma (IFNγ) to directly act on T cells to engage them in generating, shaping, and maintaining adaptive immune response (28). The present study found that an increase in the proportion of NK cells inhibited the progression of DLBCL. It has even been found that the development of resistance to rituximab, the first-line drug used to treat DLBCL, is also inextricably linked to abnormal NK cell counts (29). This finding is consistent with the current study (30).

Increases in the metabolites Kynurenine levels and 2’-o-methylcytidine levels increased the risk of FL, and increases in 1-methylxanthine levels and dihydroferulate levels decreased the risk of FL. It has been found that kynurenine is associated with high levels of inflammation and is involved in the abnormal regulation of endocrine, metabolic, and hormonal systems (31). All of the above provide theoretical hypotheses that kynurenine levels increase the risk of FL. An increase in the ratio of the immunocyte phenotype CD28 on CD39+ secreting Treg increases the risk of FL.

Increases in Octanoylcarnitine (c8) levels, Linoleate (18:2n6) levels, and levels of the unknown metabolite X-15728 decreased the risk of MCL and increases in Arachidonate (20:4n6) to linoleate (18:2n6) increased the risk of MCL. It has been established that obesity is associated with the risk of developing multiple subtypes of NHL (32). L-octanoylcarnitine is an independent predictor of atherosclerosis in adults (33). Thus octanoylcarnitine (c8) levels might be a potential factor in the increased risk of HNL due to obesity. The immunocyte phenotype CD25 on transitional was positively associated with the risk of developing MCL.

Immunocyte phenotypes of CX3CR1 on CD14- CD16- and CD45 on basophil increase the risk of WM. This study did not find a significant causal relationship between metabolites and WM. Considering that the original data on WM are only available for 88 cases, the risk factors for WM need to be further explored.

Increases in Palmitate (16:0) to myristate (14:0) ratio, Glucose to maltose ratio, and ADP to glycerol 3-phosphate ratio reduced the risk of CLL. It was found that Palmitate could activate mitogen-activated protein kinases (MAPK) and then contribute to DNA damage in CLL cells (34, 35), ultimately leading to CLL cell apoptosis. The increased ADP rate reduces the risk of CLL is also consistent with previous studies (36). Unsw mem AC was positively associated with the risk of developing MCL. Increased CD86+ plasmacytoid DC AC and CD127 on CD28+ CD45RA- CD8br decreased the risk of developing CLL.

Mannose to N-acetylglucosamine to N-acetylgalactosamine ratio was negatively correlated with the risk of developing MTCL.CD4 Treg %CD4 was positively correlated with the risk of developing MTCL, a result that is consistent with the findings of conventional studies. A high Treg ratio will inhibit T cells from exerting immune activity, leading to an increased risk of MTCL, which is characterized by abnormalities in the T cell system (37).

Increases in the unnamed metabolites X-21258 levels and Propionylglycine levels decreased the risk of HL, and increases in the AMP to IMP ratio increased the risk of HL. No significant correlation between immunocyte phenotypes and HL was found in this study. This study did not find a significant bidirectional causal relationship between metabolites or immunocyte phenotypes and MZL.

In summary, we have found a strong link between metabolites and lymphoma. However, there is no established mechanism by which metabolites affect lymphomas. Follow-up studies have shown that CD27 on PB/PC may be an intermediate mediator of the positive correlation between Arginine to glutamate ratio and CLL risk. NK %lymphocyte is an intermediate mediator of the negative correlation of the unknown metabolite X-18922 levels with DLBCL. CD25 on IgD- CD24- is an intermediate mediator of the negative association of unknown metabolite X-24531 levels with DLBCL. Kara IO et al. found that sCD27 was an independent prognostic factor in the assessment of CLL (38), consistent with the findings of this study. NK cells are derived from bone marrow hematopoietic stem cells and represent an essential component of the intrinsic immune system (27). NK cells not only act as killer cells to suppress tumors, but NK cells can also use IFNγ to act directly on T cells to engage them in generating, shaping, and maintaining adaptive immune responses (28). Thus the percentage of NK cells to lymphocytes can represent a mediator of metabolite-negative regulation of lymphomas.

So far, we are the first to explore the causal relationship between metabolites, immunocyte phenotypes, and lymphoma risk by MR, and demonstrated that some immunocyte phenotypes are mediators between metabolites and lymphoma. However, there are still some flaws in this study: (1) Three immunocyte phenotypes were horizontally pleiotropic when analyzed with lymphoma MR, requiring replacement of the database for further research. (2) Possibly due to differences in the study population and investigators, five immunocyte phenotypes were heterogeneous when analyzed with lymphoma MR. (3) We screened for IVs using a p-value of P less than 1 × 10^-5^, so the IVs were not strongly correlated enough, although they did allow for a more comprehensive assessment of the association between metabolites, immune cell phenotypes, and lymphoma. (4) Insufficient SNP data on WM. When WM is used as an exposure factor, the thresholds for the selected P-values are different from those for other subtypes of lymphoma. The above may lead to the conclusion that WM is not equivalent compared to other subtypes of lymphoma. (5) Although the GWAS for metabolite data selected for this study focused more on individuals of European ancestry, the cases selected were Canadian. In addition, SNPs associated with human immune cell phenotypes were referenced from the Sardinian population, an isolated, homogeneous island population. The GWAS pooled data for eight types of lymphomas from Finngen (https://www.FinnGen.fi/en/) which has a population with multiple known population bottlenecks and was isolated from the general European population. In addition, the two-sample Mendelian randomization analysis method has limitations when dealing with multiple exposures. Therefore, there is a need to explore suitable analytical methods. Finally, to draw clinical conclusions, we also need to conduct comprehensive clinical trials for validation; therefore, a more comprehensive GWAS database and further analytical methods or experimental validation are required to elucidate the relationship between individual metabolites, immune cell phenotypes, and lymphomas as well as their impact mechanisms.

## Conclusion

Here in this study, we comprehensively explored the causal relationship between metabolites, immunocyte phenotypes, and lymphoma. We found that 17 metabolites were causally associated with lymphoma in both directions, and 12 immunocyte phenotypes were causally associated with lymphoma in both directions. We also identified causal relationships between metabolites and lymphomas, with three groups mediated by immunocyte phenotypes. In addition, we identified a causal relationship between metabolites and lymphomas, with three groups mediated by immunocyte phenotypes. We could inhibit disease progression by interfering with the expression of CD25, NK cells, and CD27 in future studies of DLBCL and CLL, respectively, with a multiplier effect. These are potential research opportunities.

## Data Availability

The original contributions presented in the study are included in the article/**Supplementary Material**. Further inquiries can be directed to the corresponding author/s.

## References

[1] Lewis WD Lilly S Jones KL. Lymphoma: diagnosis and treatment. Am Fam Physician. (2020) 101:34–41. 31894937

[2] Armitage JO Gascoyne RD Lunning MA Cavalli F. Non-Hodgkin lymphoma. Lancet. (2017) 390:298–310. doi: 10.1016/S0140-6736(16)32407-2 28153383

[3] Chen Y Lu T Pettersson-Kymmer U Stewart ID Butler-Laporte G Nakanishi T. Genomic atlas of the plasma metabolome prioritizes metabolites implicated in human diseases. Nat Genet. (2023) 55:44–53. doi: 10.1038/s41588-022-01270-1 36635386 PMC7614162

[4] Pietzner M Stewart ID Raffler J Khaw K-T Michelotti GA Kastenmüller G. Plasma metabolites to profile pathways in noncommunicable disease multimorbidity. Nat Med. (2021) 27:471–9. doi: 10.1038/s41591-021-01266-0 PMC8127079 33707775

[5] Lotta LA Pietzner M Stewart ID Wittemans LBL Li C Bonelli R. A cross-platform approach identifies genetic regulators of human metabolism and health. Nat Genet. (2021) 53:54–64. doi: 10.1038/s41588-020-00751-5 33414548 PMC7612925

[6] Csizmar CM Ansell SM. Engaging the innate and adaptive antitumor immune response in lymphoma. Int J Mol Sci. (2021) 22:3302. doi: 10.3390/ijms22073302 33804869 PMC8038124

[7] Mancuso S Mattana M Carlisi M Santoro M Siragusa S. Effects of B-cell lymphoma on the immune system and immune recovery after treatment: the paradigm of targeted therapy. Int J Mol Sci. (2022) 23:3368. doi: 10.3390/ijms23063368 35328789 PMC8952275

[8] Cronin SJF Seehus C Weidinger A Talbot S Reissig S Seifert M. The metabolite BH4 controls T cell proliferation in autoimmunity and cancer. Nature. (2018) 563:564–8. doi: 10.1038/s41586-018-0701-2 PMC6438708 30405245

[9] Moon J-Y Zolnik CP Wang Z Qiu Y Usyk M Wang T. Gut microbiota and plasma metabolites associated with diabetes in women with, or at high risk for, HIV infection. EBioMedicine. (2018) 37:392–400. doi: 10.1016/j.ebiom.2018.10.037 30366816 PMC6286648

[10] Oliveira L de M Teixeira FME Sato MN. Impact of retinoic acid on immune cells and inflammatory diseases. Mediators Inflamm. (2018) 2018:3067126. doi: 10.1155/2018/3067126 30158832 PMC6109577

[11] Xia L Oyang L Lin J Tan S Han Y Wu N. The cancer metabolic reprogramming and immune response. Mol Cancer. (2021) 20:28. doi: 10.1186/s12943-021-01316-8 33546704 PMC7863491

[12] Yuan X Ouedraogo SY Trawally M Tan Y Bajinka O. Cancer energy reprogramming and the immune responses. Cytokine. (2024) 177:156561. doi: 10.1016/j.cyto.2024.156561 38430694

[13] Chen K-C Hsiao I-H Huang Y-N Chou Y-T Lin Y-C Hsieh J-Y. Targeting human mitochondrial NAD(P)+-dependent Malic enzyme (ME2) impairs energy metabolism and redox state and exhibits antileukemic activity in acute myeloid leukemia. Cell Oncol (Dordr). (2023) 46:1301–16. doi: 10.1007/s13402-023-00812-x PMC10618384 37079187

[14] Li J Li C Huang Y Guan P Huang D Yu H. Mendelian randomization analyses in ocular disease: a powerful approach to causal inference with human genetic data. J Transl Med. (2022) 20:621. doi: 10.1186/s12967-022-03822-9 36572895 PMC9793675

[15] Gupta V Walia GK Sachdeva MP. Mendelian randomization”: an approach for exploring causal relations in epidemiology. Public Health. (2017) 145:113–9. doi: 10.1016/j.puhe.2016.12.033 28359378

[16] Davies NM Holmes MV Davey Smith G. Reading Mendelian randomisation studies: a guide, glossary, and checklist for clinicians. BMJ. (2018) 362:k601. doi: 10.1136/bmj.k601 30002074 PMC6041728

[17] Orrù V Steri M Sidore C Marongiu M Serra V Olla S. Complex genetic signatures in immune cells underlie autoimmunity and inform therapy. Nat Genet. (2020) 52:1036–45. doi: 10.1038/s41588-020-0684-4 PMC8517961 32929287

[18] Burgess S Thompson SG. Interpreting findings from Mendelian randomization using the MR-Egger method. Eur J Epidemiol. (2017) 32:377–89. doi: 10.1007/s10654-017-0255-x PMC5506233 28527048

[19] Bowden J Davey Smith G Haycock PC Burgess S. Consistent estimation in Mendelian randomization with some invalid instruments using a weighted median estimator. Genet Epidemiol. (2016) 40:304–14. doi: 10.1002/gepi.21965 PMC4849733 27061298

[20] Higgins JPT Thompson SG Deeks JJ Altman DG. Measuring inconsistency in meta-analyses. BMJ. (2003) 327:557–60. doi: 10.1136/bmj.327.7414.557 PMC192859 12958120

[21] Yavorska OO Burgess S. MendelianRandomization: an R package for performing Mendelian randomization analyses using summarized data. Int J Epidemiol. (2017) 46:1734–9. doi: 10.1093/ije/dyx034 PMC5510723 28398548

[22] Rodríguez C Puente-Moncada N Reiter RJ Sánchez-Sánchez AM Herrera F Rodríguez-Blanco J. Regulation of cancer cell glucose metabolism is determinant for cancer cell fate after melatonin administration. J Cell Physiol. (2021) 236:27–40. doi: 10.1002/jcp.29886 32725819

[23] Callao V Montoya E. Toxohormone-like factor from microorganisms with impaired respiration. Science. (1961) 134:2041–2. doi: 10.1126/science.134.3495.2041 13875778

[24] Zhang J Yang J Lin C Liu W Huo Y Yang M. Endoplasmic Reticulum stress-dependent expression of ERO1L promotes aerobic glycolysis in Pancreatic Cancer. Theranostics. (2020) 10:8400–14. doi: 10.7150/thno.45124 PMC7381747 32724477

[25] Rui R Zhou L He S. Cancer immunotherapies: advances and bottlenecks. Front Immunol. (2023) 14:1212476. doi: 10.3389/fimmu.2023.1212476 37691932 PMC10484345

[26] Scott DW Gascoyne RD. The tumour microenvironment in B cell lymphomas. Nat Rev Cancer. (2014) 14:517–34. doi: 10.1038/nrc3774 25008267

[27] O’Brien KL Finlay DK. Immunometabolism and natural killer cell responses. Nat Rev Immunol. (2019) 19:282–90. doi: 10.1038/s41577-019-0139-2 30808985

[28] Vivier E Rebuffet L Narni-Mancinelli E Cornen S Igarashi RY Fantin VR. Natural killer cell therapies. Nature. (2024) 626:727–36. doi: 10.1038/s41586-023-06945-1 38383621

[29] Cox MC Battella S La Scaleia R Pelliccia S Di Napoli A Porzia A. Tumor-associated and immunochemotherapy-dependent long-term alterations of the peripheral blood NK cell compartment in DLBCL patients. Oncoimmunology. (2015) 4:e990773. doi: 10.4161/2162402X.2014.990773 25949906 PMC4404844

[30] de Jonge AV Duetz C Bruins WSC Korst CLBM Rentenaar R Cosovic M. Distinct peripheral T-cell and NK-cell profiles in HGBL-MYC/BCL2 vs patients with DLBCL NOS. Blood Adv. (2024) 8:1094–104. doi: 10.1182/bloodadvances.2023011687 PMC10907399 38191686

[31] Savitz J. The kynurenine pathway: a finger in every pie. Mol Psychiatry. (2020) 25:131–47. doi: 10.1038/s41380-019-0414-4 PMC6790159 30980044

[32] Maskarinec G Brown SM Lee J Bogumil D Walsh C Haiman CA. Association of obesity and type 2 diabetes with non-Hodgkin lymphoma: the multiethnic cohort. Cancer Epidemiol Biomarkers Prev. (2023) 32:1348–55. doi: 10.1158/1055-9965.EPI-23-0565 PMC10592150 37555836

[33] Kim M Jung S Lee S-H Lee JH. Association between arterial stiffness and serum L-octanoylcarnitine and lactosylceramide in overweight middle-aged subjects: 3-year follow-up study. PloS One. (2015) 10:e0119519. doi: 10.1371/journal.pone.0119519 25781947 PMC4363527

[34] Wang S Gu J Xu Z Zhang Z Bai T Xu J. Zinc rescues obesity-induced cardiac hypertrophy *via* stimulating metallothionein to suppress oxidative stress-activated BCL10/CARD9/p38 MAPK pathway. J Cell Mol Med. (2017) 21:1182–92. doi: 10.1111/jcmm.13050 PMC5431126 28158919

[35] Ecker V Brandmeier L Stumpf M Giansanti P Moreira AV Pfeuffer L. Negative feedback regulation of MAPK signaling is an important driver of chronic lymphocytic leukemia progression. Cell Rep. (2023) 42:113017. doi: 10.1016/j.celrep.2023.113017 37792532

[36] Bellosillo B Piqué M Barragán M Castaño E Villamor N Colomer D. Aspirin and salicylate induce apoptosis and activation of caspases in B-cell chronic lymphocytic leukemia cells. Blood. (1998) 92:1406–14. doi: 10.1182/blood.V92.4.1406 9694730

[37] Bittner S Hehlgans T Feuerer M. Engineered Treg cells as putative therapeutics against inflammatory diseases and beyond. Trends Immunol. (2023) 44:468–83. doi: 10.1016/j.it.2023.04.005 37100644

[38] Kara IO Sahin B Gunesacar R. Expression of soluble CD27 and interleukins-8 and -10 in B-cell chronic lymphocytic leukemia: correlation with disease stage and prognosis. Adv Ther. (2007) 24:29–40. doi: 10.1007/BF02849990 17526459

