# Supplementary material for: Metabolite, immunocyte phenotype, and lymphoma: a Mendelian randomization study
Source: Front Immunol. 2024 Sep 25;15:1431261. doi: 10.3389/fimmu.2024.1431261 (PMC11461196; doi:10.3389/fimmu.2024.1431261)
Supplement: Supplementary file 1 [file DataSheet1.pdf]

Additional Figures

Figure S1. MR leave-one-out sensitivity analysis for Metabolite on DLBCL.

Figure S2. MR leave-one-out sensitivity analysis for Metabolite on FL.

Figure S3. MR leave-one-out sensitivity analysis for Metabolite on MCL.

Figure S4. MR leave-one-out sensitivity analysis for Metabolite on CLL.

Figure S5. MR leave-one-out sensitivity analysis for Metabolite on T/NK CL.

Figure S6. MR leave-one-out sensitivity analysis for Metabolite on HL.

Figure S7. Scatter plots for the effect of Metabolite on DLBCL.

Figure S8. Scatter plots for the effect of Metabolite on FL.

Figure S9. Scatter plots for the effect of Metabolite on MCL.

Figure S10. Scatter plots for the effect of Metabolite on CLL.

Figure S11. Scatter plots for the effect of Metabolite on T/NK CL.

Figure S12. Scatter plots for the effect of Metabolite on HL.

Figure S13. Forest plots for the effect of Metabolite on DLBCL.

Figure S14. Forest plots for the effect of Metabolite on FL.

Figure S15. Forest plots for the effect of Metabolite on MCL.

Figure S16. Forest plots for the effect of Metabolite on CLL.

Figure S17. Forest plots for the effect of Metabolite on T/NK CL.

Figure S18. Forest plots for the effect of Metabolite on HL.

Figure S1. MR leave-one-out sensitivity analysis for Metabolite on DLBCL.

(A)
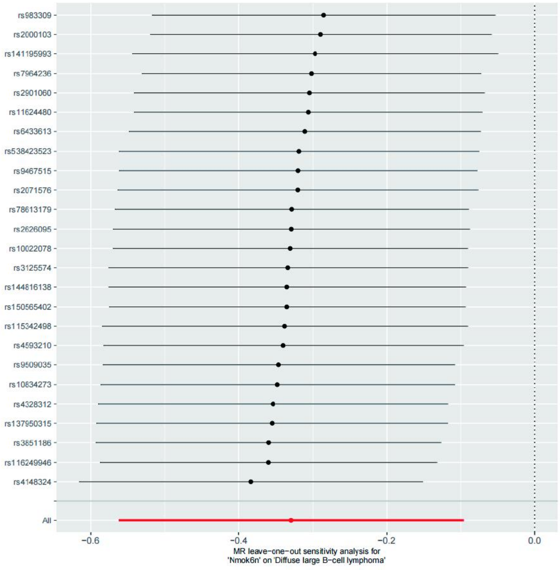

(B)
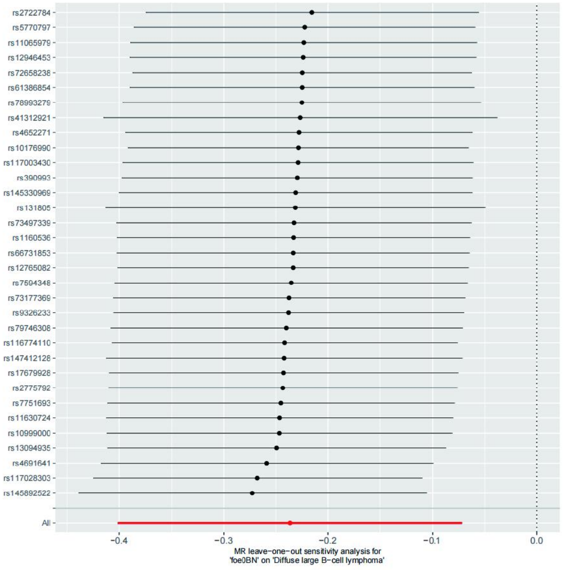

(A) Analysis for "X-11632 levels,id.GCST90200466" on "DLBCL"

(B) Analysis for "Phosphate to 2'-deoxyuridine ratio,id.GCST90200768" on "DLBCL"

Figure S2. MR leave-one-out sensitivity analysis for Metabolite on FL.

(A)

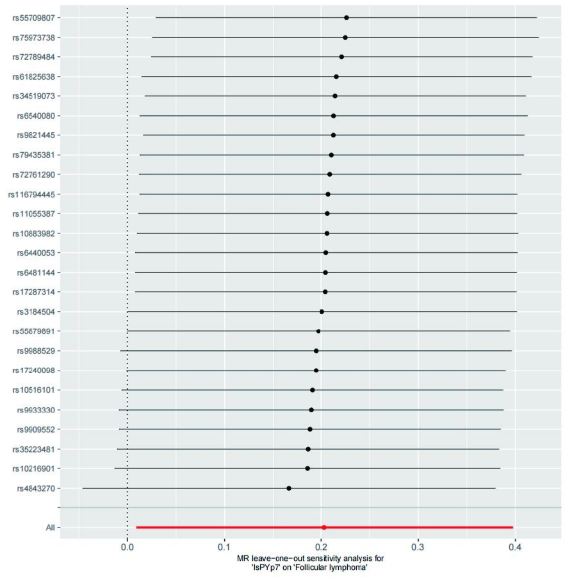

(B)

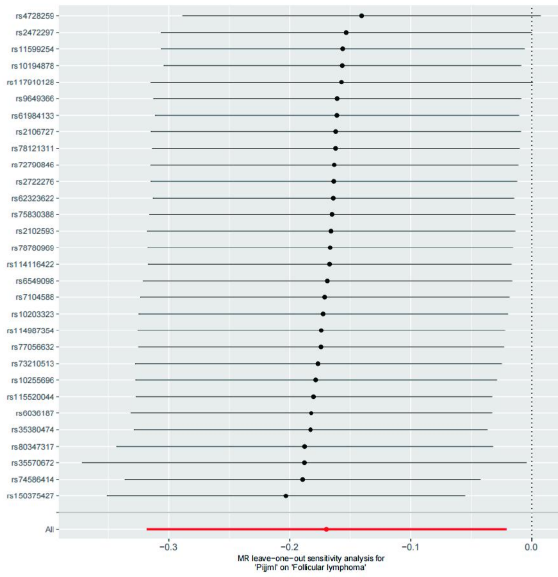

(C)

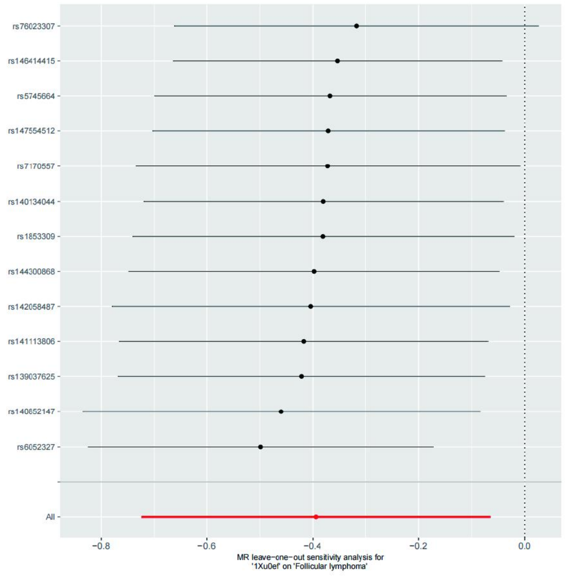

(D)

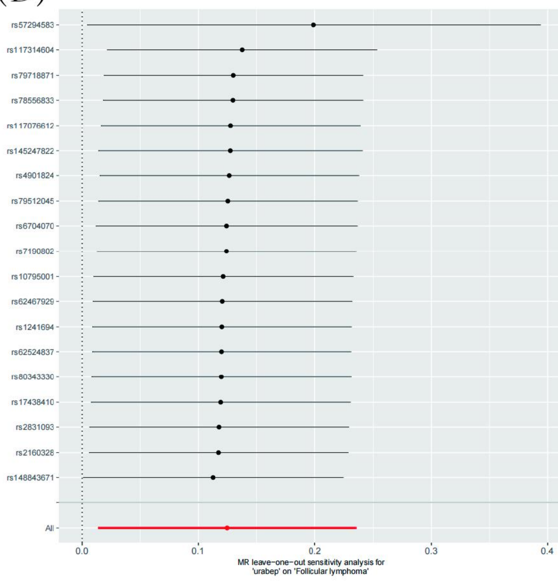

(A) Analysis for "Kynurenine levels,id.GCST90199636" on "FL"
(B) Analysis for "1-methylxanthine levels,id.GCST90199763" on "FL"
(C) Analysis for "Dihydroferulate levels,id.GCST90199921" on "FL"
(D) Analysis for "2'-o-methylcytidine levels,id.GCST90200694" on "FL"

Figure S3. MR leave-one-out sensitivity analysis for Metabolite on MCL.

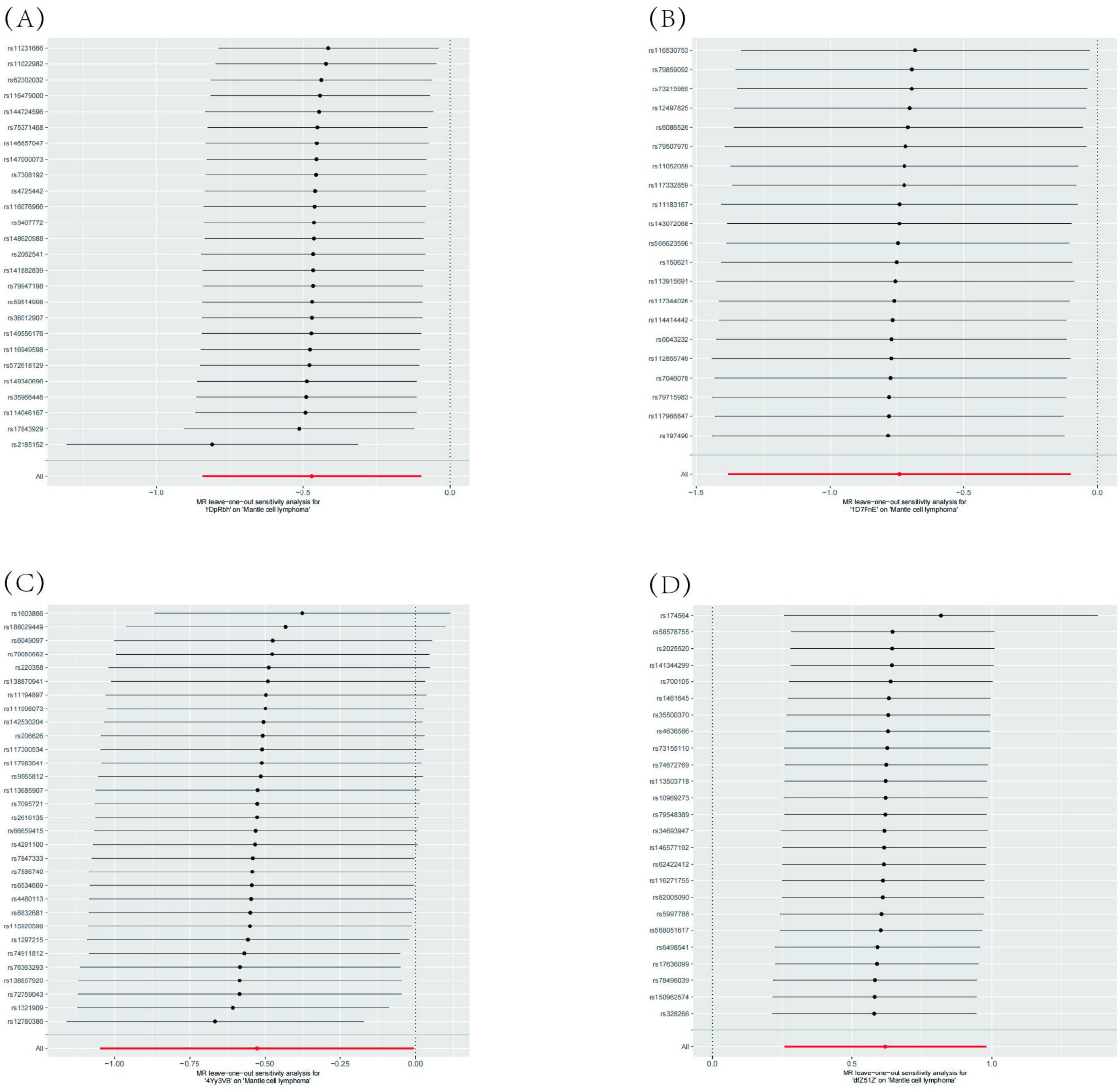

(A) Analysis for "Octanoylcarnitine (c8) levels,id.GCST90199720" on "MCL"
(B) Analysis for "Linoleate (18:2n6) levels,id.GCST90200354" on "MCL"
(C) Analysis for "X-15728 levels,id.GCST90200542" on "MCL"
(D) Analysis for "Arachidonate (20:4n6) to linoleate (18:2n6) ratio,id.GCST90200979" on "MCL"

Figure S4. MR leave-one-out sensitivity analysis for Metabolite on CLL.

(A)

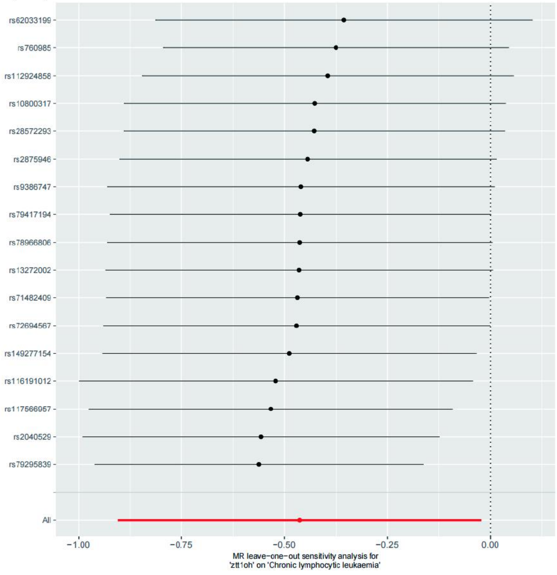

(B)

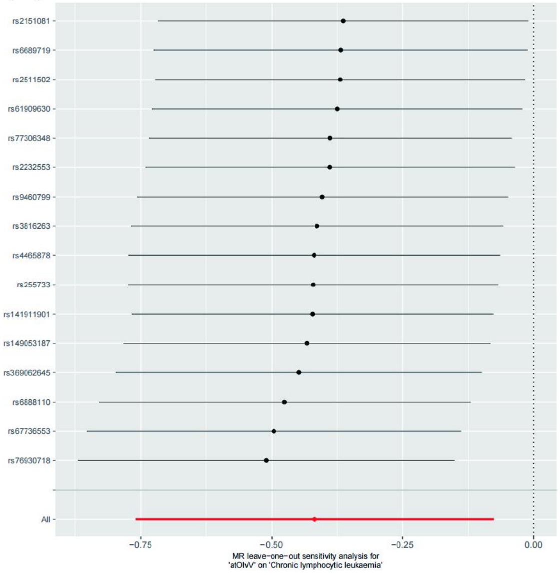

(C)

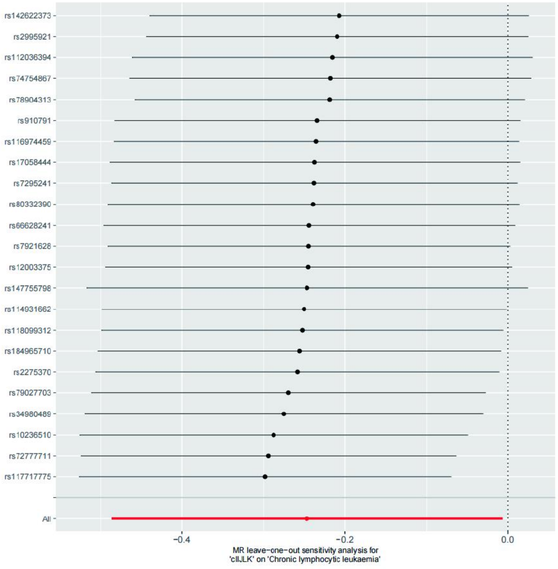

(A) Analysis for "Palmitate (16:0) to myristate (14:0) ratio,id.GCST90200762" on "CLL"

(B) Analysis for "Glucose to maltose ratio,id.GCST90200781" on "CLL"

(C) Analysis for "Adenosine 5'-diphosphate (ADP) to glycerol 3-phosphate ratio,id.GCST90200834" on "CLL"

Figure S5. MR leave-one-out sensitivity analysis for Metabolite on T/NK CL.

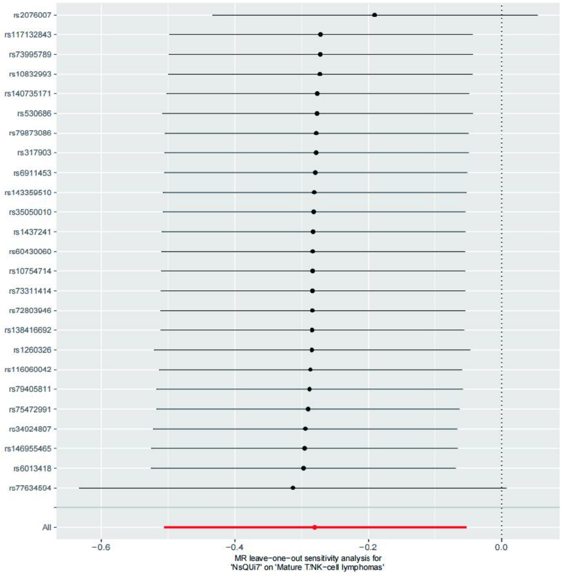

Analysis for "Mannose to N-acetylglucosamine to N-acetylgalactosamine ratio,id.GCST90200884" on "T/NK CL"

Figure S6. MR leave-one-out sensitivity analysis for Metabolite on HL.

(A)

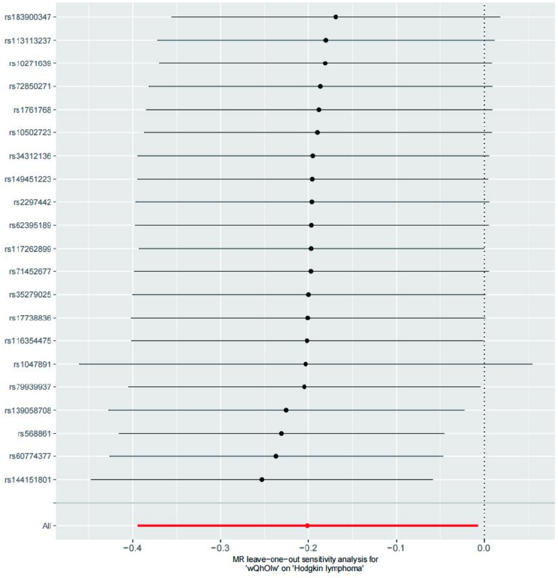

(B)

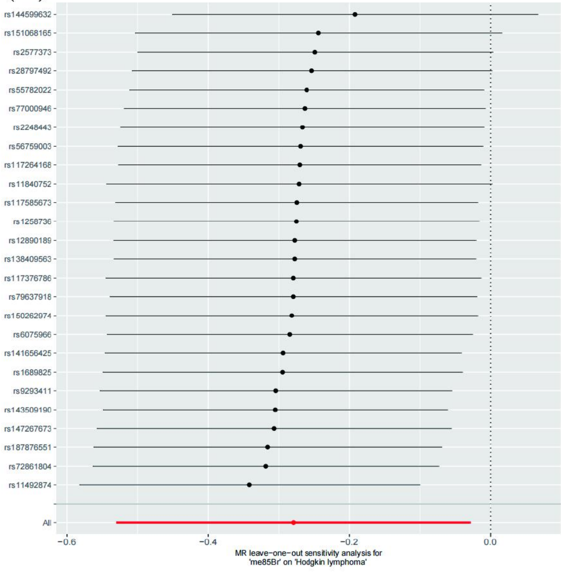

(C)

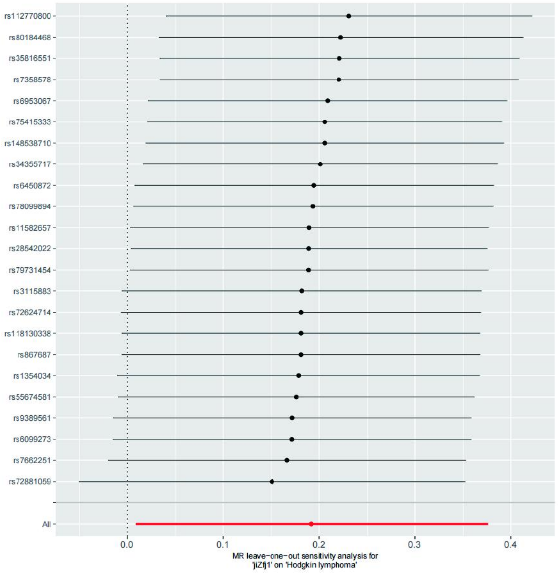

(A) Analysis for "Propionylglycine levels,id.GCST90199705" on "HL"
(B) Analysis for "X-21258 levels,id.GCST90200565" on "HL"
(C) Analysis for "AMP to IMP ratio,id.GCST90200738" on "HL"

Figure S7. Scatter plots for the effect of Metabolite on DLBCL.

(A)

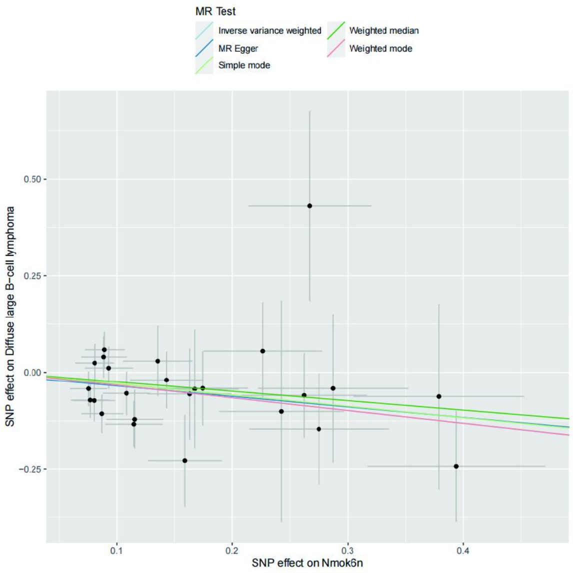

(B)

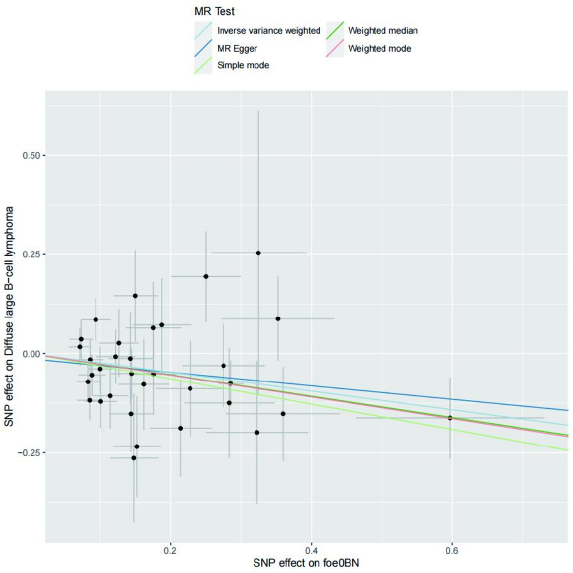

(A)Analysis for "X-11632 levels,id.GCST90200466" on "DLBCL"

(B) Analysis for "Phosphate to 2'-deoxyuridine ratio,id.GCST90200768" on "DLBCL"

Figure S8. Scatter plots for the effect of Metabolite on DLBCL.

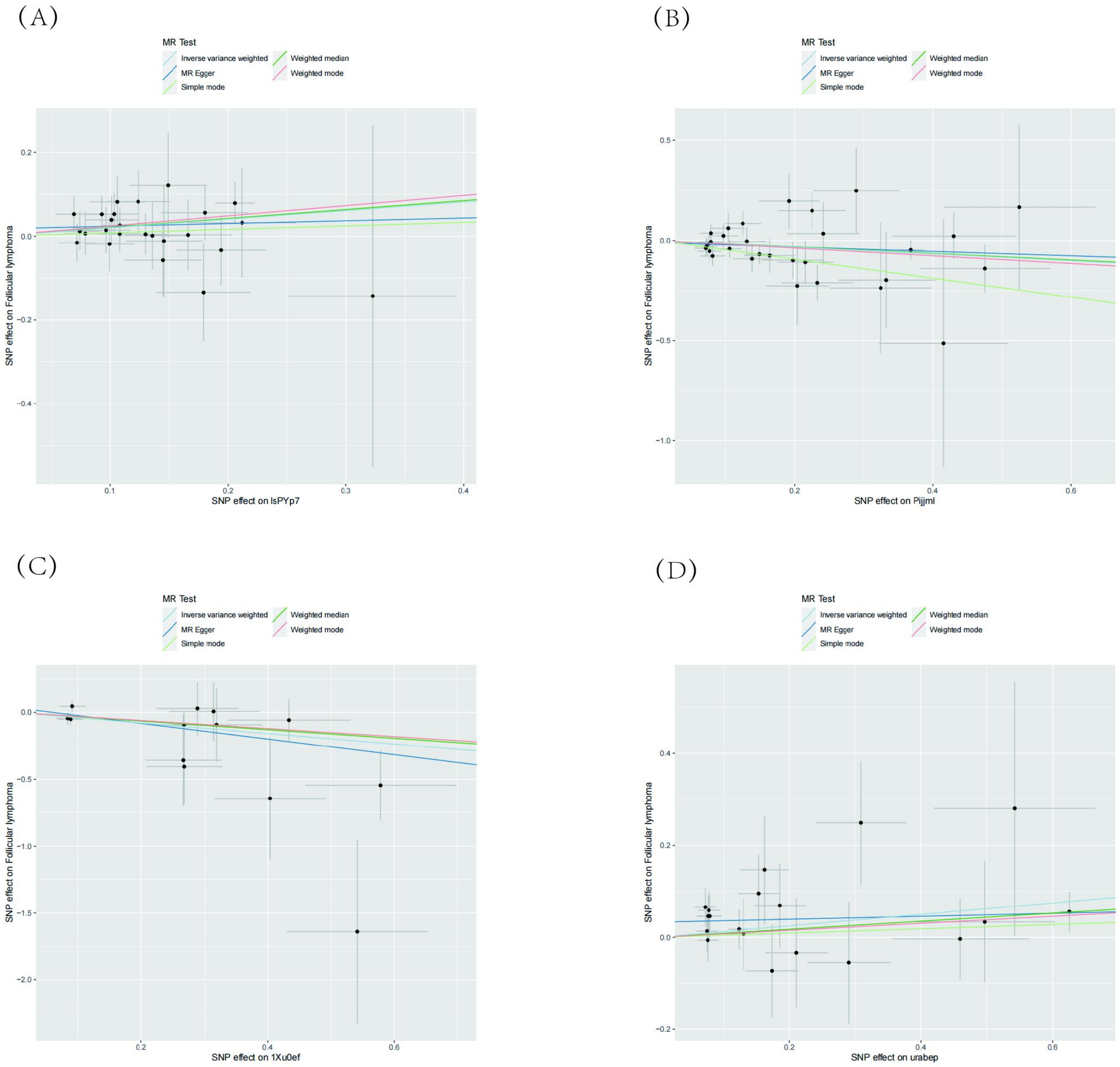

(A) Analysis for "Kynurenine levels,id.GCST90199636" on "FL"
(B) Analysis for "1-methylxanthine levels,id.GCST90199763" on "FL"
(C) Analysis for "Dihydroferulate levels,id.GCST90199921" on "FL"
(D) Analysis for "2'-o-methylcytidine levels,id.GCST90200694" on "FL"

Figure S9. Scatter plots for the effect of Metabolite on MCL.

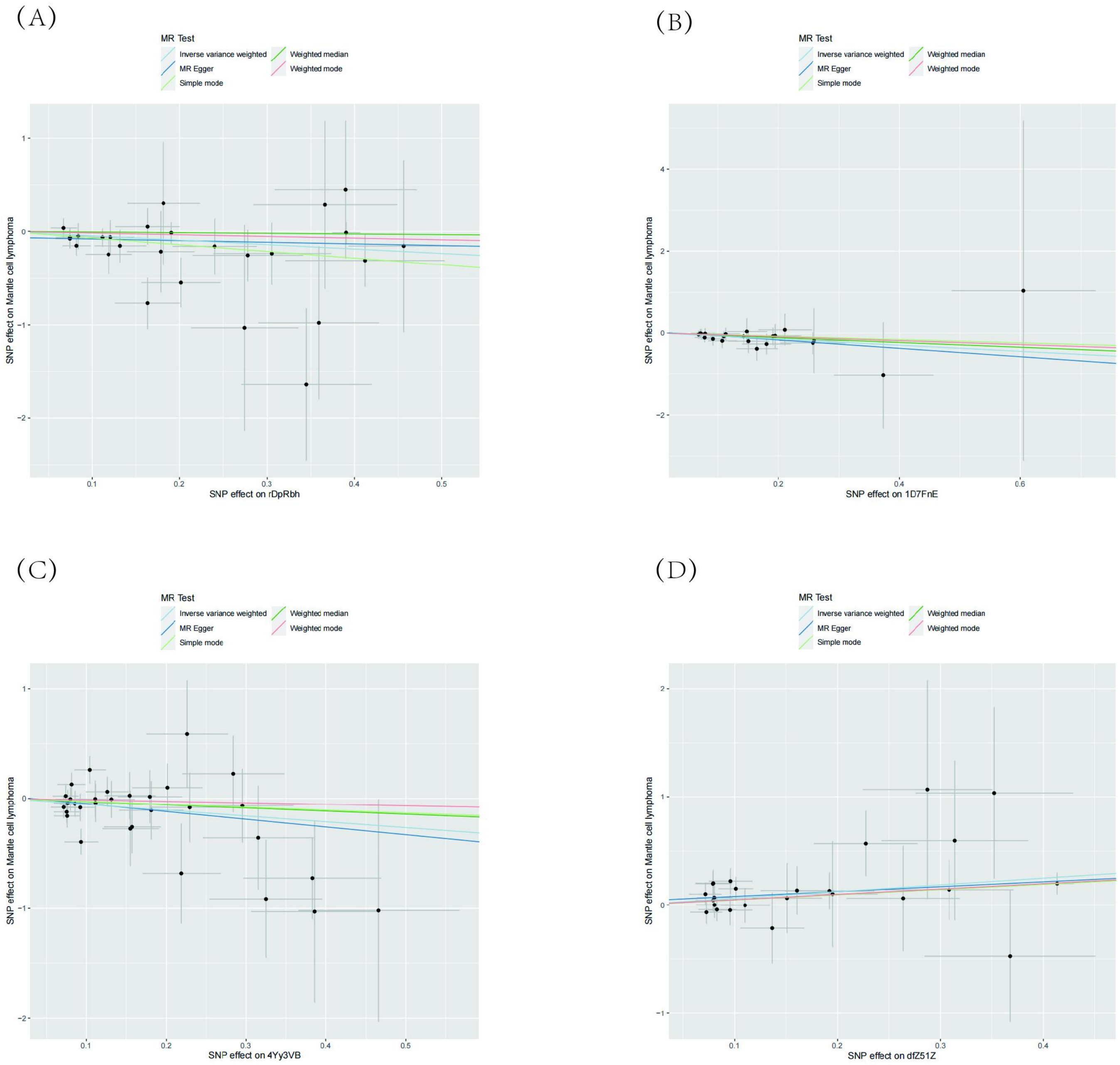

(A) Analysis for "Octanoylcarnitine (c8) levels,id.GCST90199720" on "MCL"
(B) Analysis for "Linoleate (18:2n6) levels,id.GCST90200354" on "MCL"
(C) Analysis for "X-15728 levels,id.GCST90200542" on "MCL"
(D) Analysis for "Arachidonate (20:4n6) to linoleate (18:2n6) ratio,id.GCST90200979" on "MCL"

Figure S10. Scatter plots for the effect of Metabolite on CLL.

(A)

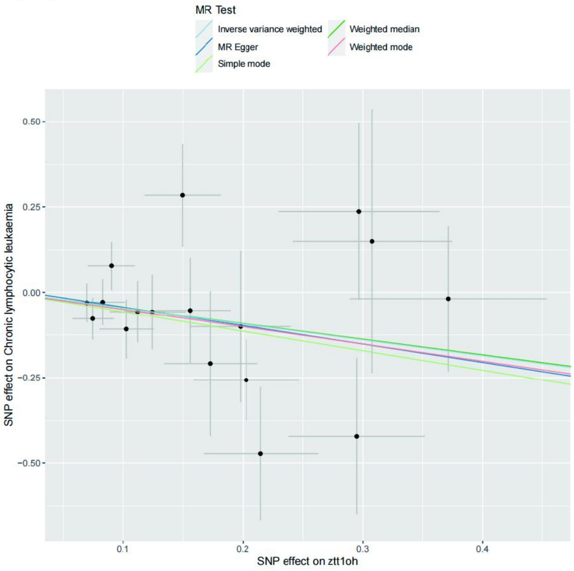

(B)

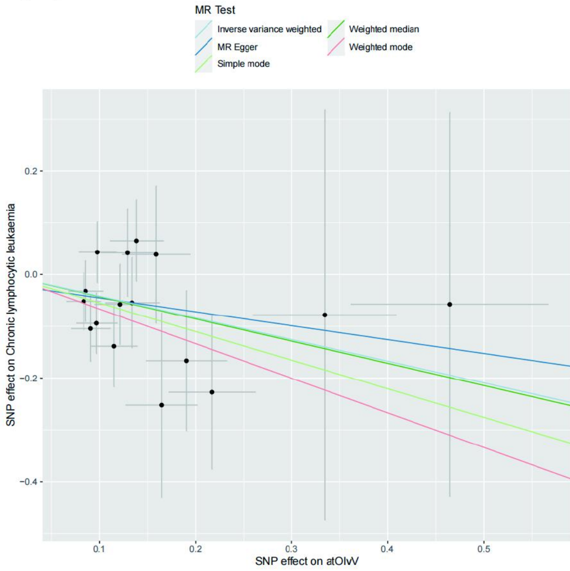

(C)

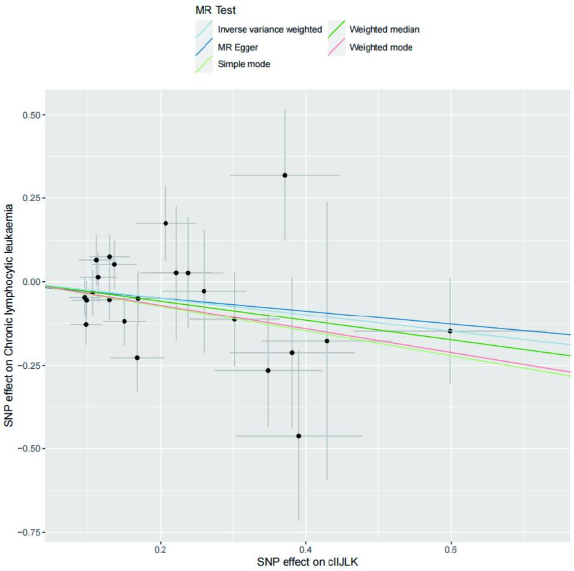

(A) Analysis for "Palmitate (16:0) to myristate (14:0) ratio,id.GCST90200762" on "CLL"

(B) Analysis for "Glucose to maltose ratio,id.GCST90200781" on "CLL"

(C) Analysis for "Adenosine 5'-diphosphate (ADP) to glycerol 3-phosphate ratio,id.GCST90200834" on "CLL"

Figure S11. Scatter plots for the effect of Metabolite on T/NK CL.

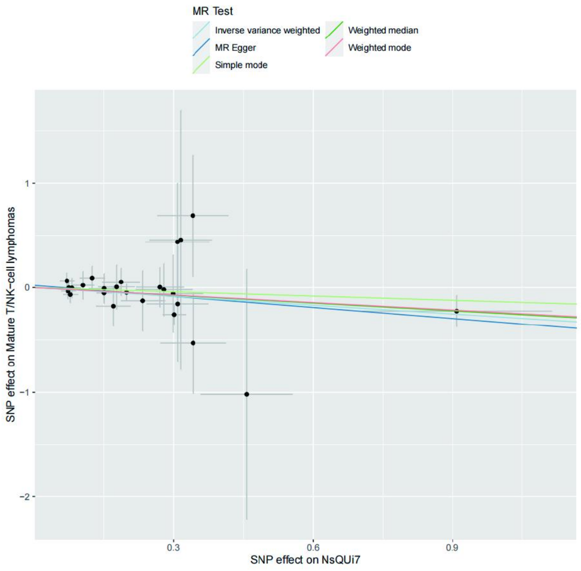

Analysis for "Mannose to N-acetylglucosamine to N-acetylgalactosamine ratio,id.GCST90200884" on "T/NK CL"

Figure S12. Scatter plots for the effect of Metabolite on HL.

(A)

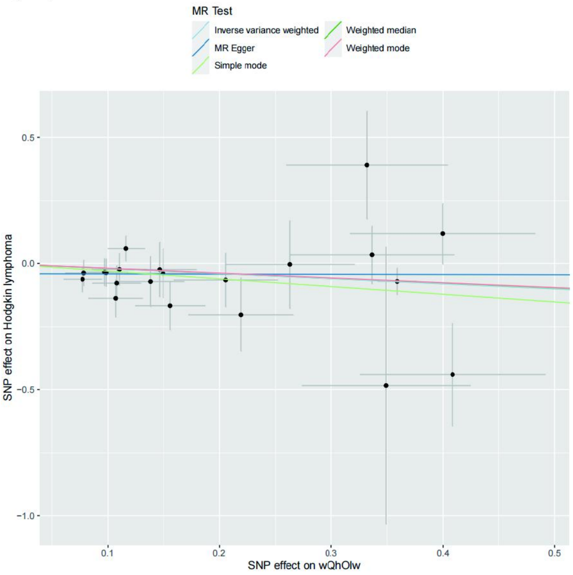

(B)

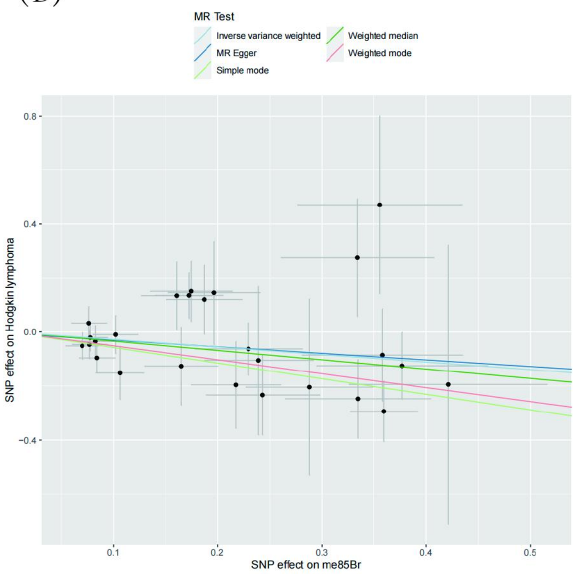

(C)

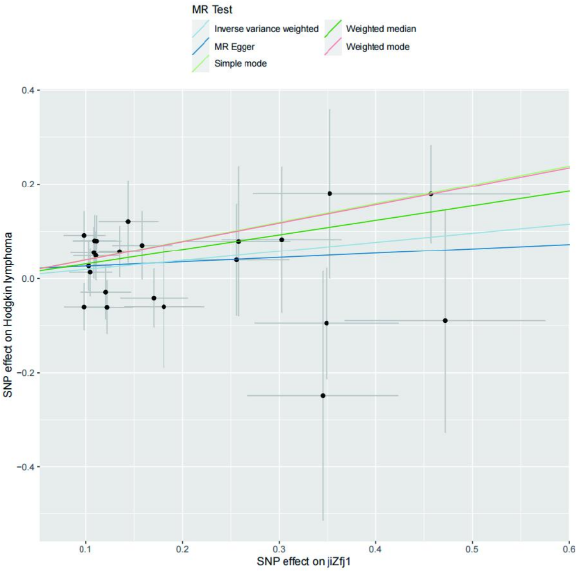

(A) Analysis for "Propionylglycine levels,id.GCST90199705" on "HL"
(B) Analysis for "X-21258 levels,id.GCST90200565" on "HL"
(C) Analysis for "AMP to IMP ratio,id.GCST90200738" on "HL"

Figure S13. Forest plots for the effect of Metabolite on DLBCL.

(A)

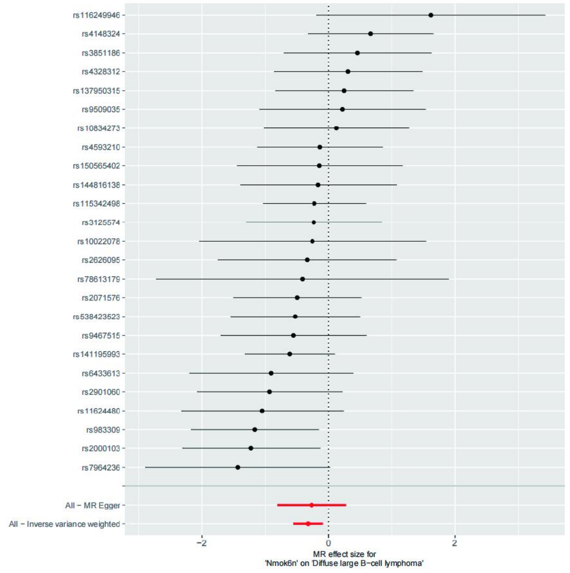

(B)

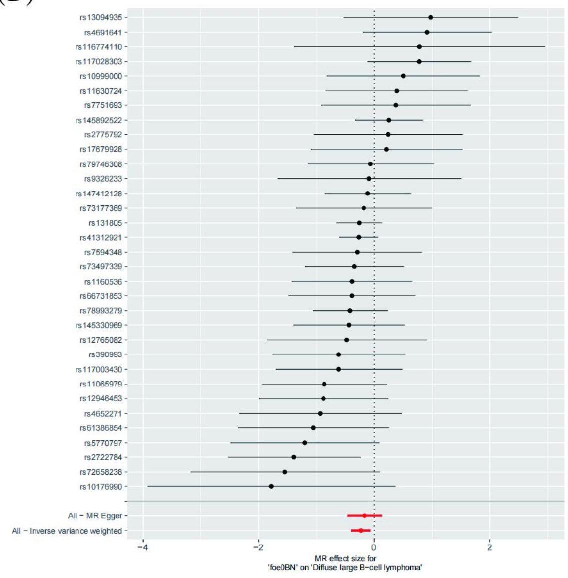

(A) MR effect size for "X-11632 levels,id.GCST90200466" on "DLBCL"

(B) MR effect size for "Phosphate to 2'-deoxyuridine ratio,id.GCST90200768" on "DLBCL"

Figure S14. Forest plots for the effect of Metabolite on FL.

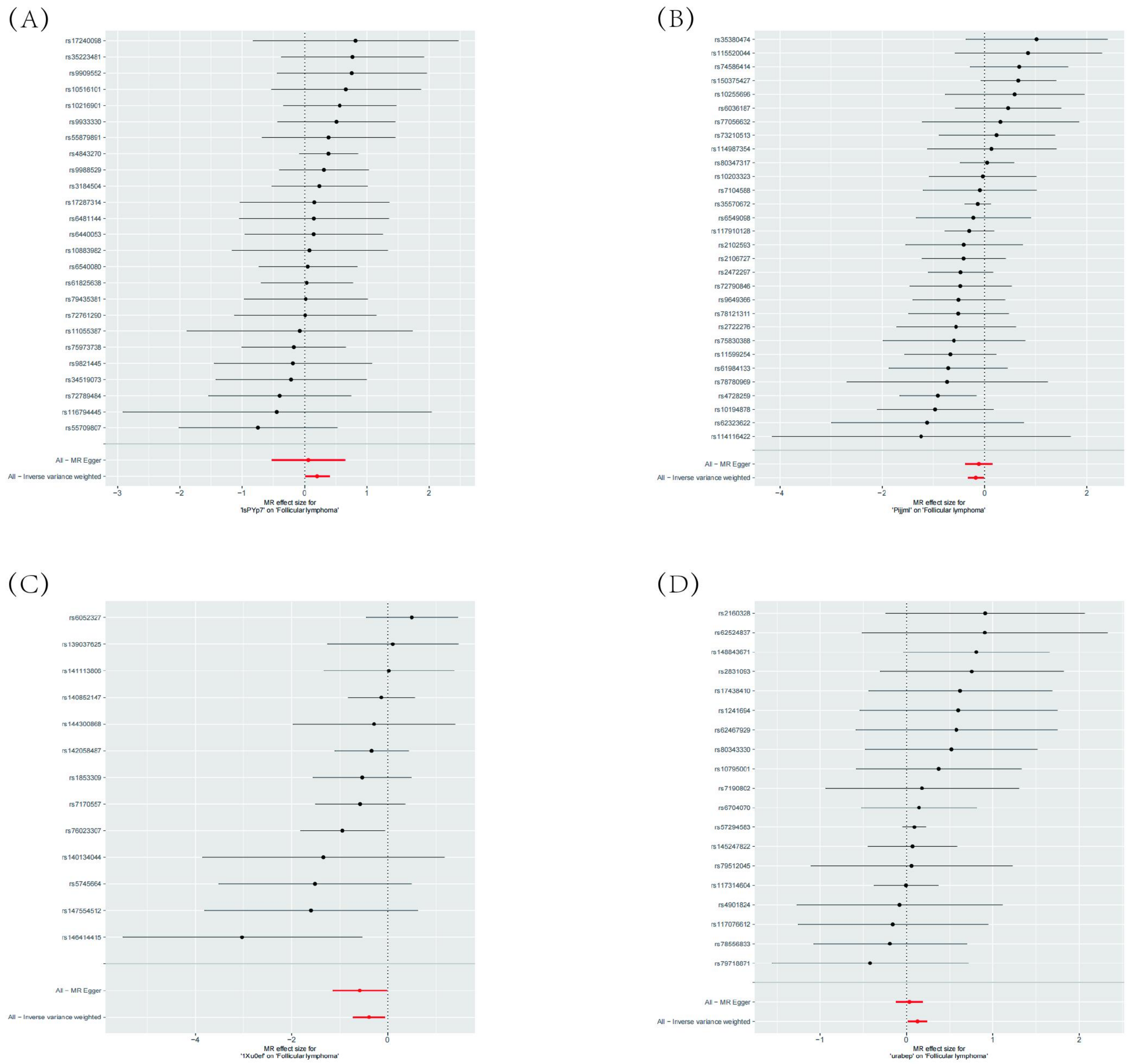

(A) MR effect size for "Kynurenine levels,id.GCST90199636" on "FL"

(B) MR effect size for "1-methylxanthine levels,id.GCST90199763" on "FL"

(C) MR effect size for "Dihydroferulate levels,id.GCST90199921" on "FL"

(D) MR effect size for "2'-o-methylcytidine levels,id.GCST90200694" on "FL"

Figure S15. Forest plots for the effect of Metabolite on MCL.

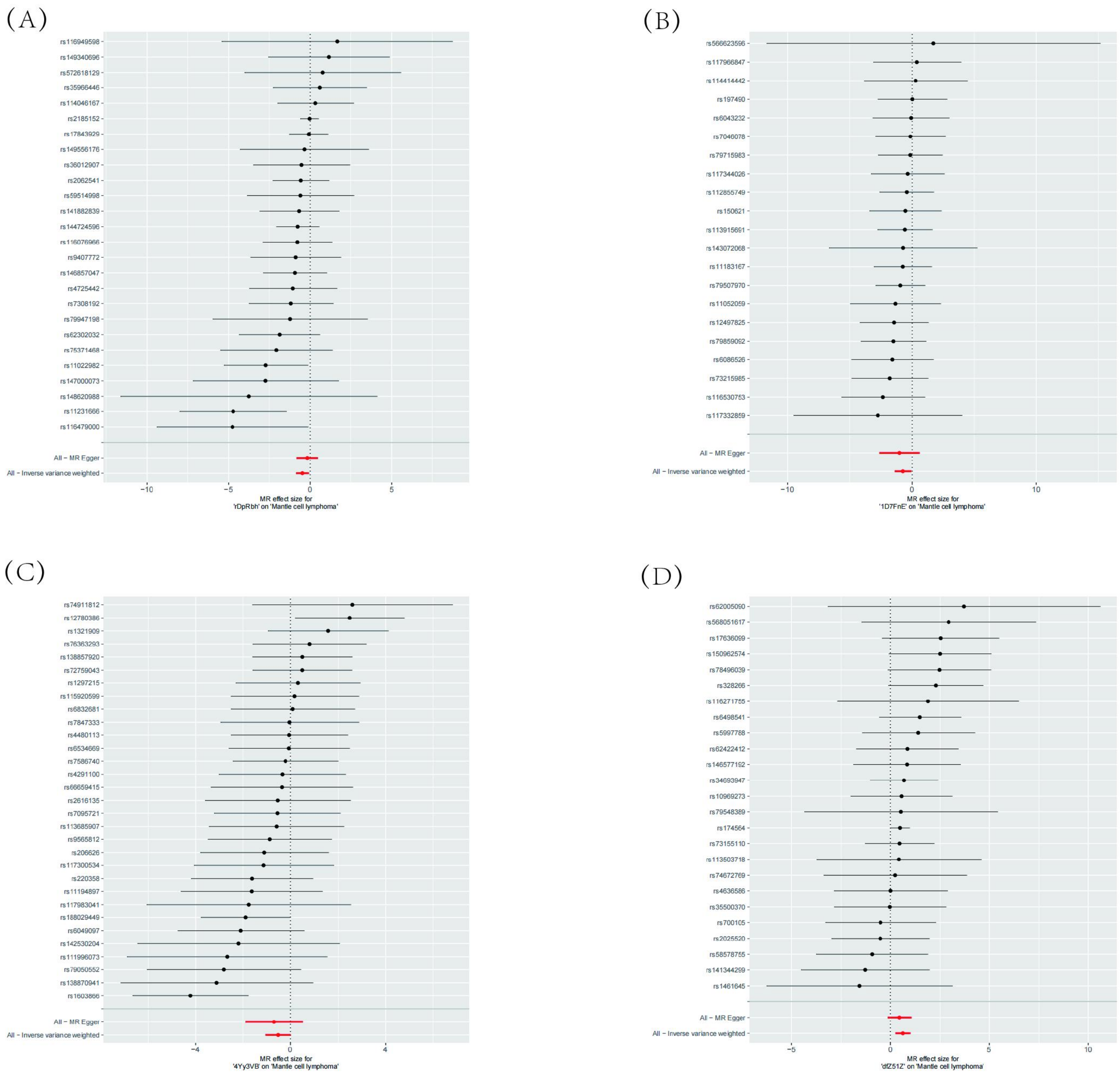

(A) MR effect size for "Octanoylcarnitine (c8) levels,id.GCST90199720" on "MCL"
(B) MR effect size for "Linoleate (18:2n6) levels,id.GCST90200354" on "MCL"
(C) MR effect size for "X-15728 levels,id.GCST90200542" on "MCL"
(D) MR effect size for "Arachidonate (20:4n6) to linoleate (18:2n6) ratio,id.GCST90200979" on "MCL"

Figure S16. Forest plots for the effect of Metabolite on CLL.

(A)

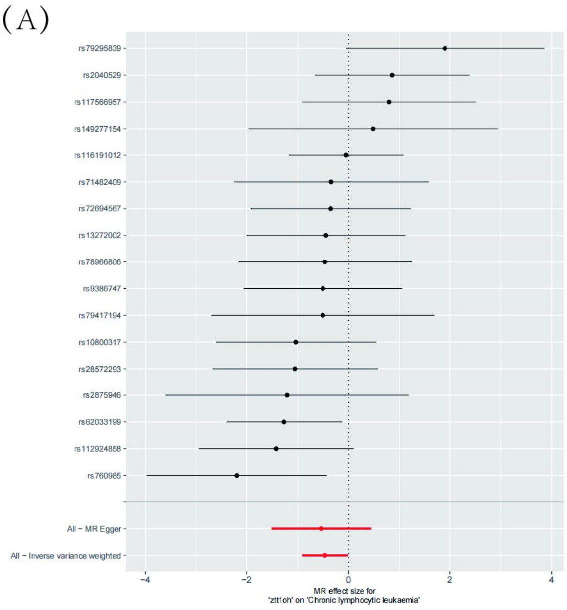

(B)

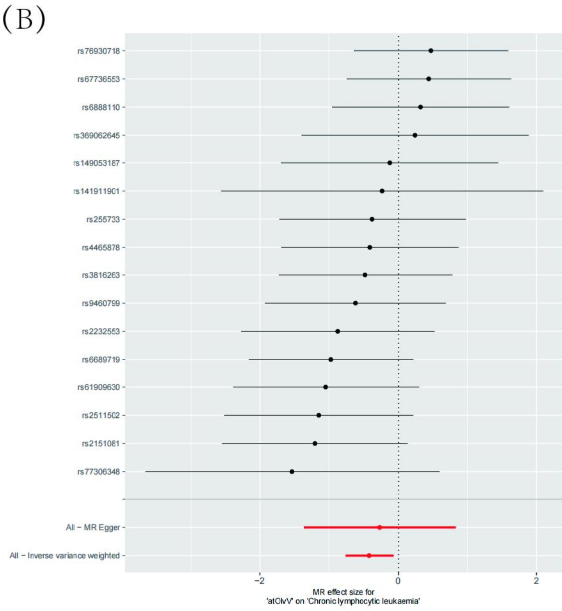

(C)

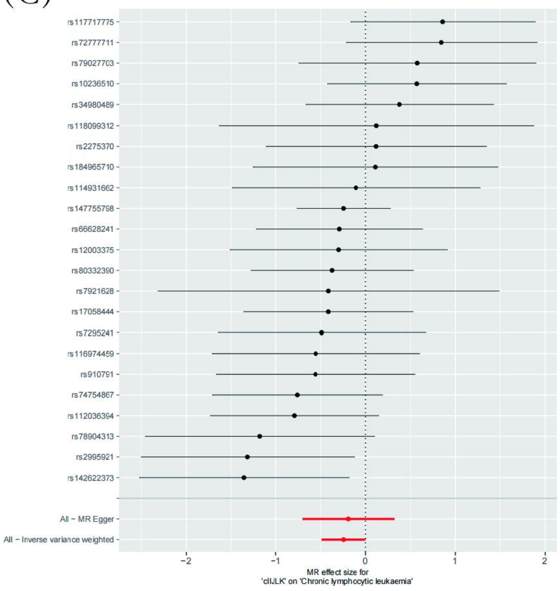

(A) MR effect size for "Palmitate (16:0) to myristate (14:0) ratio,id.GCST90200762" on "CLL"

(B) MR effect size for "Glucose to maltose ratio,id.GCST90200781" on "CLL"

(C) MR effect size for "Adenosine 5'-diphosphate (ADP) to glycerol 3-phosphate ratio,id.GCST90200834" on "CLL"

Figure S17. Forest plots for the effect of Metabolite on T/NK CL.

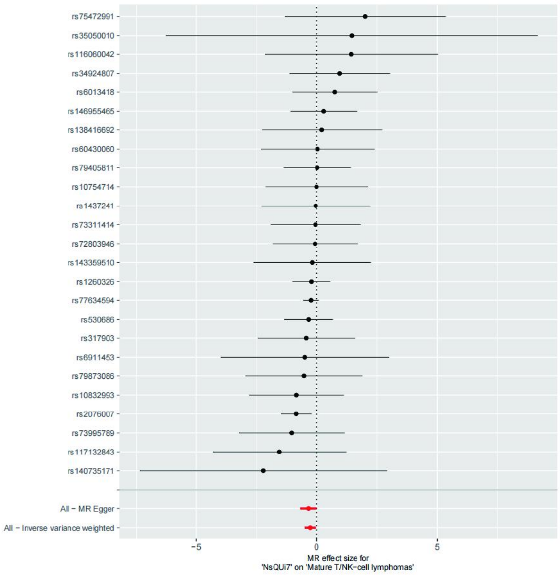

MR effect size for "Mannose to N-acetylglucosamine to N-acetylgalactosamine ratio,id.GCST90200884" on "T/NK CL"

Figure S18. Forest plots for the effect of Metabolite on HL.

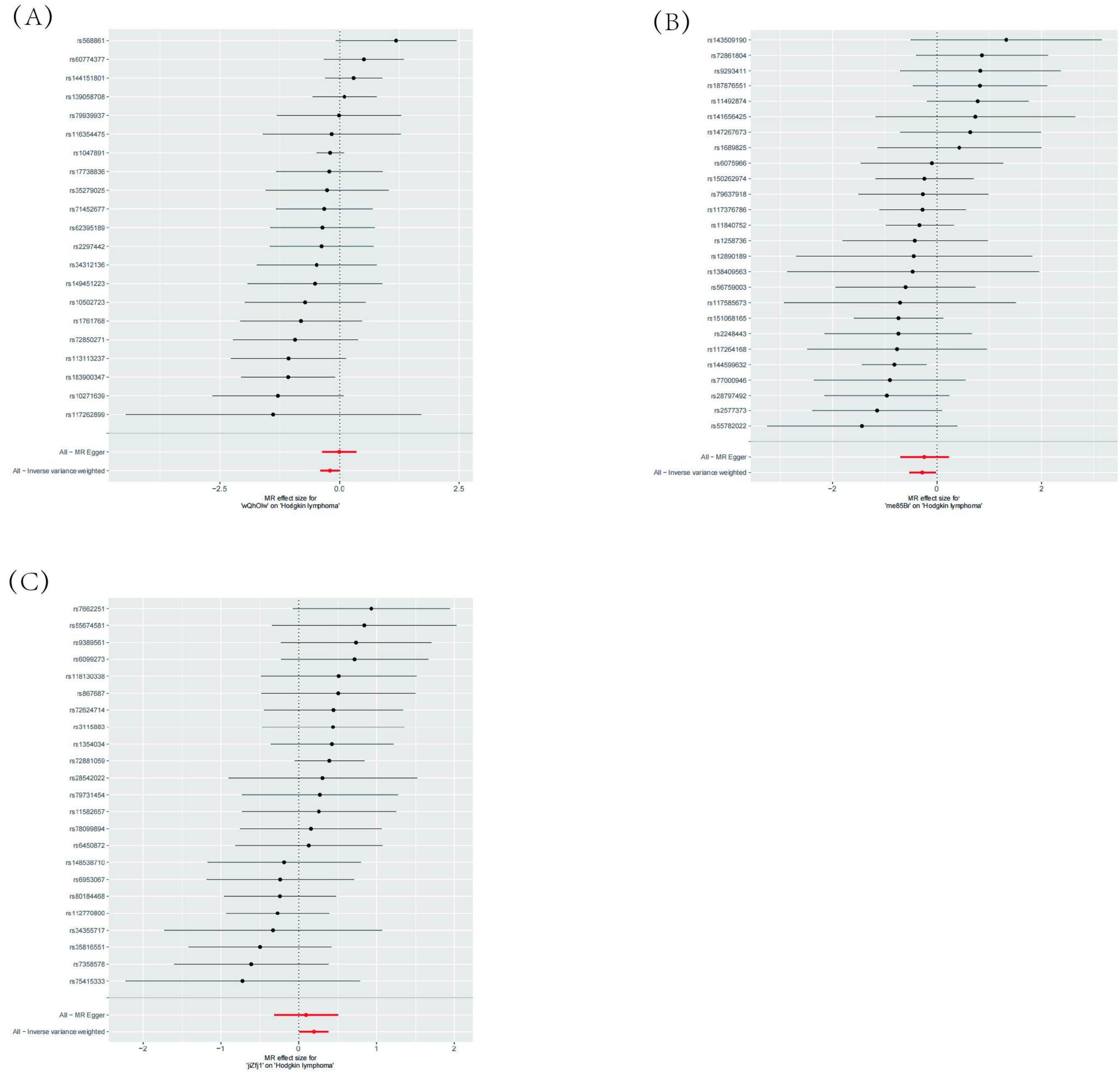

(A) MR effect size for "Propionylglycine levels,id.GCST90199705" on "HL"
(B) MR effect size for "X-21258 levels,id.GCST90200565" on "HL"
(C) MR effect size for "AMP to IMP ratio,id.GCST90200738" on "HL"
